# Single-mutation fitness landscapes for an enzyme on multiple substrates reveal specificity is globally encoded

**DOI:** 10.1038/ncomms15695

**Published:** 2017-06-06

**Authors:** Emily E. Wrenbeck, Laura R. Azouz, Timothy A. Whitehead

**Affiliations:** 1 Department of Chemical Engineering and Materials Science, Michigan State University, Engineering Building, 428 S. Shaw Lane, Room 2100, East Lansing, Michigan 48824, USA; 2 Department of Biosystems and Agricultural Engineering, Michigan State University, Farrall Hall, 524 S. Shaw Lane, Room 216, East Lansing, Michigan 48824, USA

## Abstract

Our lack of total understanding of the intricacies of how enzymes behave has constrained our ability to robustly engineer substrate specificity. Furthermore, the mechanisms of natural evolution leading to improved or novel substrate specificities are not wholly defined. Here we generate near-comprehensive single-mutation fitness landscapes comprising >96.3% of all possible single nonsynonymous mutations for hydrolysis activity of an amidase expressed in *E. coli* with three different substrates. For all three selections, we find that the distribution of beneficial mutations can be described as exponential, supporting a current hypothesis for adaptive molecular evolution. Beneficial mutations in one selection have essentially no correlation with fitness for other selections and are dispersed throughout the protein sequence and structure. Our results further demonstrate the dependence of local fitness landscapes on substrate identity and provide an example of globally distributed sequence-specificity determinants for an enzyme.

## Abstract (web summary)

Systematically understanding the sequence determinants to substrate specificity for enzymes has implications in areas from evolutionary biology to biocatalysis. Here, Whitehead and colleagues generate and analyse near-comprehensive single-mutation fitness landscapes for an amidase with three different substrates.

Understanding the sequence determinants to substrate specificity for enzymes is a significant challenge in protein science that impacts fields as diverse as evolutionary biology and biocatalysis^1^,^2^. The dynamic relationship between protein structure and function makes it difficult to predict perturbations to the primary sequence that will improve or alter activity for a given substrate^2^. More fundamental concerns relate the nature of protein fitness landscapes to a biophysical basis underlying molecular evolution and adaptation^3^,^4^. What is the distribution of fitness effects (DFE) for mutations, and do they correspond with existing theory of adaptation^5^,^6^,^7^? Are the DFE of mutations correlated between substrates^8^? Are specificity-modulating mutations correlated to bulk properties of enzymes (for example, distance to active site)?

Over the past 20 years directed evolution experiments have provided a number of insights to the above questions^9^,^10^,^11^. For engineering enzyme specificity, it has been shown that a rational mutagenesis approach—primarily focused on residues lining a substrate binding pocket—provides greater payoffs than random mutagenesis (that is, error-prone PCR)^1^,^12^,^13^. However, it is no secret that distant (>10 Å) mutations can have significant effects on catalytic function^13^,^14^,^15^,^16^,^17^,^18^,^19^,^20^. For example, in a classic paper Oue *et al*.^20^ evolved the specificity of an aspartate aminotransferase to valine and found only one mutation in direct contact with the substrate out of seventeen accumulated in the final construct. However, the spatial distribution of specificity-modulating substitutions is still unclear, as typical experiments assay the effects of less than 100 mutations. Large scale mutational studies, such as deep mutational scanning to generate local fitness landscapes^21^,^22^, provide a more comprehensive purview and can potentially be used to resolve the above open questions^23^.

From the protein engineer's perspective, the ability to predict fitness effects would greatly improve the discovery rate of beneficial mutations. In recent years, theoretical work on adaptive molecular evolution has experienced a revolution with the availability of new experimental tools. Recognizing the rare nature of beneficial mutations, Gillespie^24^ borrowed extreme value theory mathematics to predict that the DFEs for beneficial mutations, drawn from the extreme tail of DFEs, would be of the Gumbel or ‘typical' type (exponential, gamma, Weibull, and so on). Orr^6^ later proposed that beneficial mutations from a high fitness parent should be roughly exponentially distributed. While generally providing support for these theories, discerning the parameterization of a mathematical model from experimental data has yielded mixed conclusions as summarized by Orr^25^.

To explore the question of how enzymes encode specificity and scrutinize adaptive molecular evolution theory, we evaluate the sequence determinants to substrate specificity for an enzyme by generating comprehensive single-mutation fitness landscapes—the effects of all possible single point mutations—on multiple substrates. As a model system we use the aliphatic amide hydrolase encoded by *amiE* from *Pseudomonas aeruginosa*^26^ because the structure is solved^27^, amidases are an industrially-relevant class of enzymes^28^,^29^, and amiE has activity against multiple substrates. In particular, amiE maintains comparatively higher activity on acetamide and propionamide compared with the bulkier isobutyramide. Thus, our experimental system allows comparison of adaptation between similar and structurally dissimilar substrates.

## Results

### Local fitness landscapes of amiE on multiple substrates

**Figure 1:**
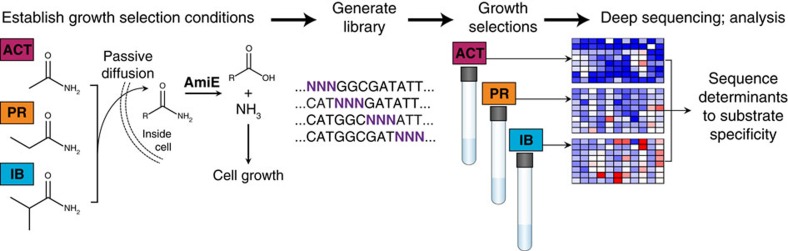
Experimental overview. Growth selections for acetamide (ACT), propionamide (PR) and isobutyramide (IB) were established. Amides passively diffuse into host cells harbouring amiE variants that produce ammonia necessary for cell growth. Comprehensive site-saturation mutagenesis libraries of amiE were made and selected in media containing an amide as the sole nitrogen source. The pre- and post-selection populations from each selection were deep sequenced and each variant was assigned a fitness metric (*ζ*) value.

**Figure 2:**
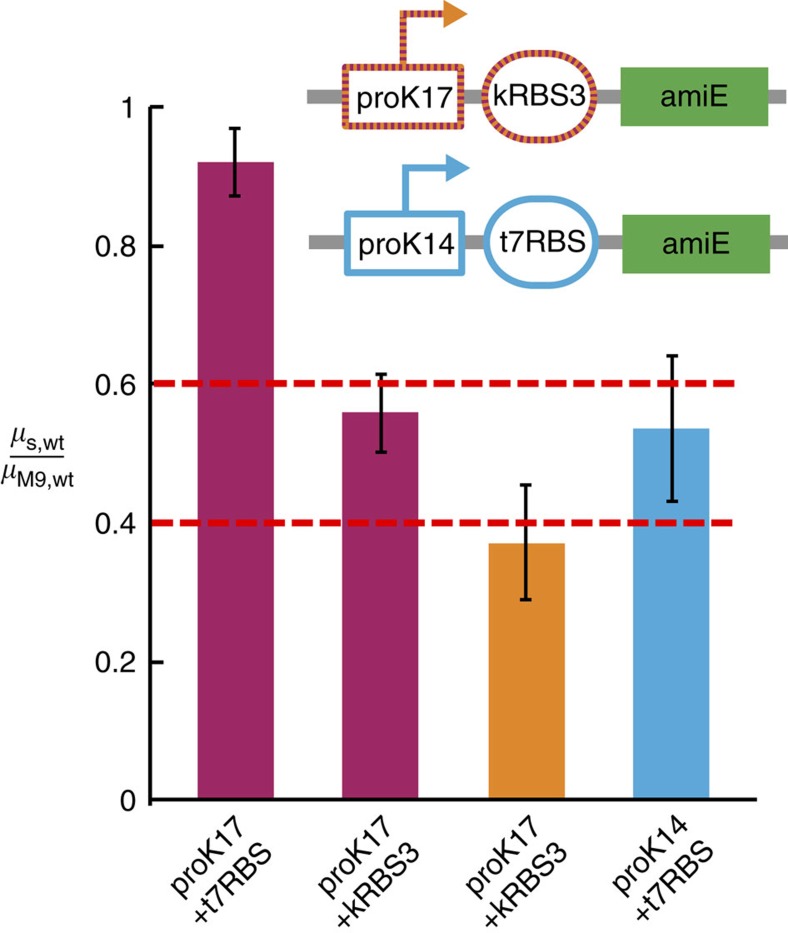
Establishing growth-based selection conditions. amiE expression was tuned using promoter and RBS engineering. *μ*_S,wt_/*μ*_M9.wt_ is the ratio of growth rate of wild-type amiE harbouring cells in selection media (*μ*_S,wt_) to M9 minimal media (*μ*_M9,wt_). Error bars represent 1 s.d. of *n*=4, 12, 10 and 12 independent measurements. Inset represents the final promoter/RBS combination used for each selection.

We first developed growth selections for three short-chain aliphatic amides: acetamide (ACT), propionamide (PR), and isobutyramide (IB) (Fig. 1), such that only *E. coli* cells harbouring a functional *amiE* gene product can grow when an amide is provided as the sole nitrogen source in selective minimal growth media^30^,^31^. Following passive diffusion into cells, amiE catalyses hydrolysis of the amide to its corresponding carboxylic acid, liberating ammonium (a bioavailable nitrogen source). To allow variants supporting higher ammonium flux to become enriched in the population relative to wild-type, we tuned amiE expression levels by screening synthetic, insulated constitutive promoters^32^,^33^ such that the specific growth rate in selection media relative to that in defined minimal media (*μ*_S,wt_/*μ*_M9,wt_) is 0.4–0.6 (ref. 34). Promoter proK14 with the high translational efficiency RBS from gene 10 of T7 bacteriophage (t7RBS) had a suitable *μ*_S,wt_/*μ*_M9,wt_ at 0.54±0.11 for IB selection media (plasmid pEDA6_amiE, Fig. 2 and Supplementary Table 1). However, the weakest promoter of the set, proK17, had a *μ*_S,wt_/*μ*_M9,wt_ of 0.92±0.05 for ACT. To further decrease protein expression, plasmids containing an altered RBS were tested. One construct containing promoter proK17 and a knockdown RBS 3 (kRBS3) sequence had a *μ*_S,wt_/*μ*_M9,wt_ of 0.56±0.06 for ACT and 0.37±0.08 for PR (plasmid pEDA2_amiE, Fig. 2 and Supplementary Table 1).

A significant concern with this growth selection is the potential for cells containing a non-functional enzyme variant to propagate in a population by acquiring ammonium that has leaked into the extracellular medium. We assessed the risk of such ‘cheating' by competing wild-type amiE on the ampicillin-resistant expression construct described above (pEDA2_amiE) against a catalytic knockout, amiE_C166S, with kanamycin-resistance on an otherwise identical expression construct (pEDK2_amiE_C166S), in ACT selection medium containing no antibiotics. *E. coli* cells harbouring either pEDA2_amiE or pEDK2_amiE_C166S were mixed in equal proportion and competed for 4.12 and 4.17 generations for replicates 1 and 2, respectively. Cells from the pre- and post-selection populations were dilution plated on ampicillin and kanamycin containing plates, and the resulting colonies counted to calculate the frequency of each member in the pre- and post-selection populations. Using the fitness equations laid out in Kowalsky *et al*.^34^, the average fitness metric for the C166S mutant was −2.40±0.9 (*n*=2), close to the fitness metric expected if no cheating occurred (−2.46). Thus, we conclude that non-functional variants minimally propagate under the conditions of the selection.

Next, we used PFunkel mutagenesis^35^ to construct comprehensive single-site saturation mutagenesis amiE libraries and transformed them into *E. coli* MG1655 rph^+^. We carried out growth selections for each of the three substrates for approximately eight generations, starting with an initial population size of >6 × 10^6^ cells. Deep sequencing of the pre- and post-selection populations was used to determine a relative fitness metric (*ζ*_i_) for each amiE variant i, defined as^34^:


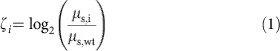


The pre-selection populations were comprised of >51.8% single nonsynonymous mutations and represented >96.3% of the 6,820 possible single nonsynonymous mutations for all libraries (Supplementary Table 2, Supplementary Fig. 1). Given that the read counts per variant in the pre-selection population were log-normally distributed (Supplementary Fig. 1) and underrepresented variants could show biased fitness metrics, we calculated Pearson's product moment correlation coefficients for pre-selection read counts and fitness and found them to be to 0.047, 0.033 and −0.064 for the ACT, PR and IB selections, respectively (Supplementary Fig. 2). This confirmed that resulting fitness metrics were not biased by a wide distribution of pre-selection read counts. Furthermore, we determined a lower bound fitness metric for each selection that can be discriminated based on depth of sequencing coverage (see Methods), such that while below this value fitness effects can be described categorically as ‘deleterious', the quantitative effect cannot be reliably predicted. The lower bounds were found to be −1.3, −0.8 and −0.6 for the ACT, PR and IB selections, respectively. Heat map representations of the local fitness landscapes for each selection can be found in Supplementary Figs 3–5.

**Figure 3:**
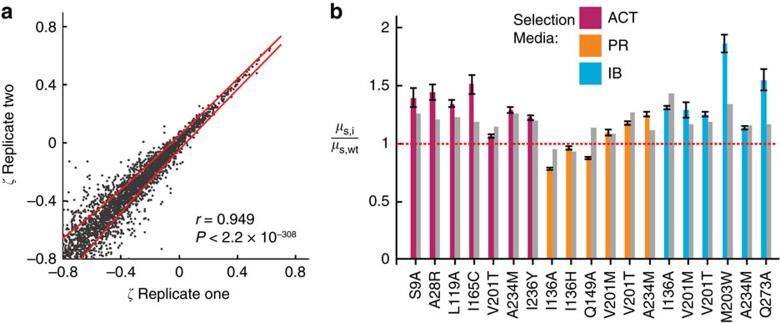
Validation of deep-sequencing results. (**a**) Fitness metrics from replicate growth selections in the propionamide selection (*n*=2,954). Red lines indicate two s.d.'s from theoretical error estimation^34^. The reported *P* value for the Pearson's product moment correlation coefficient was calculated using a two-tailed *t*-test. (**b**) Comparison of relative growth rates calculated from the selection experiments (grey bars) and isogenic growth rate assays (coloured bars). Error bars represent one s.d. of at least four independent measurements.

We tested the validity of using deep sequencing to reconstruct fitness in multiple ways. First, we performed replicate growth selections using the same pre-selection library. The resulting two post-selection libraries were prepared for sequencing in parallel attaching unique Illumina barcodes to each, and normalized fitness metrics were calculated for each replicate. To assess whether the selection results were reproducible we calculated the Pearson product moment correlation coefficients of fitness metrics between replicates and found them to be 0.661, 0.842 and 0.889 for the ACT, PR and IB selections, respectively (*P*<2.2 × 10^−308^, *n*=6,627, 6,630 and 6,569). When we excluded variants with fitness metrics below the lower bounds the correlation coefficients improved to 0.932, 0.949 and 0.943 for the ACT, PR and IB selections, respectively (*P*<2.2 × 10^−308^, *n*=3,834, 2,954, 4,977, Fig. 3a and Supplementary Fig. 6).

Second, we compared relative isogenic growth rates (*μ*_S,i_/*μ*_S,wt_) to deep sequencing-calculated growth rates for a set of mutations (Fig. 3b, Supplementary Table 3). Deep-sequencing derived fitness corresponded to increased growth rates for 16/17 beneficial mutations, near wild-type growth rates for 2/2 neutral mutations, and no growth for 1/1 deleterious mutation tested. To confirm that improved growth rates were a result of increased flux through amiE, we performed lysate activity assays for a subset of these variants and found that all samples save one improved flux relative to wild-type (Supplementary Table 3).

### The DFE of amiE

**Figure 4:**
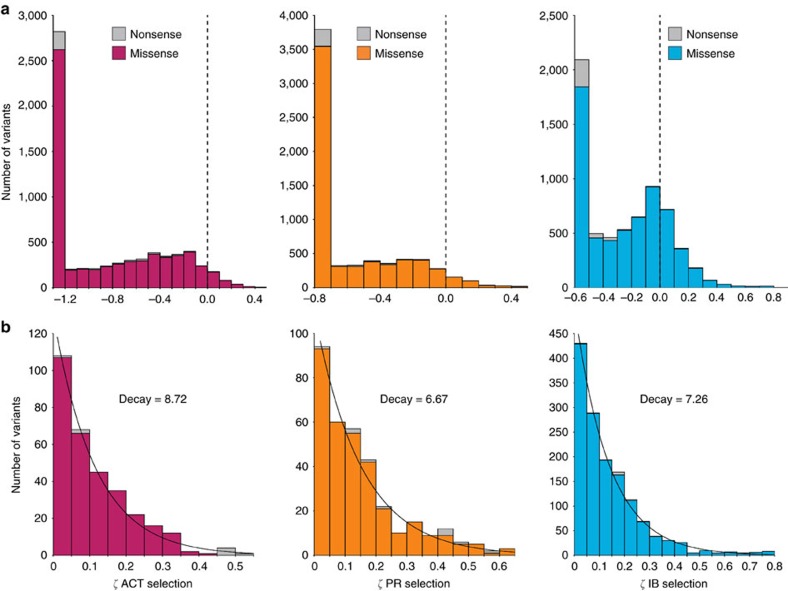
Distribution of fitness effects (DFE) are exponentially distributed for beneficial mutations. (**a**) The DFE of missense and nonsense mutations for ACT (cranberry), PR (orange) and IB (cyan) growth selections. The dashed vertical line demarcates the wild-type fitness metric (*ζ*=0.0). (**b**) DFE for beneficial mutations identified in the ACT, PR and IB selections, respectively. Overlain curves are best-fit exponential distributions estimated from the data.

The DFE, both at the organismal and protein level, demarcates evolution^5^,^7^,^36^. Specifically, the DFE for a protein is related to its evolvability: the number and type of available beneficial mutations for a new function compared with effects on existing functions is illustrative of how natural proteins evolve. While theoretical and experimental work has advanced our understanding of the available pathways for adaptive molecular evolution^4^,^6^,^37^,^38^,^39^,^40^,^41^,^42^,^43^,^44^, the exact form of the distribution, which determines these pathways, is still a subject of debate. Figure 4a shows the DFE for the three selections. For each, nonsense mutations had a median fitness metric below the detection limit of the deep sequencing method. Nonsense mutations with increased fitness metrics (*ζ*>0.15) cluster in the last 19 residues of the C terminus, a relatively unstructured region likely to have no influence on catalytic activity, suggesting that translation of these residues plus the C-terminal His_6_-tag used for purification is deleterious to fitness.

Missense mutations were on average deleterious for the ACT and PR selections, with 75.8% and 74.2% of variants yielding at least 20% reduction in growth rate relative to wild-type, respectively. By contrast, only 45.4% fell below this threshold for the IB selection. Remarkably, 21.5% (*n*=1,394) of missense mutations had above wild-type fitness metrics for the IB selection, with 483 (7.5%) variants having at least 10% increased growth rate (*ζ*>0.15). There were appreciably less enhanced variants found in the ACT and PR selections, with 4.7% and 5.1% (*n*=306 and 328) having fitness metrics above wild-type, respectively.

Modern theories of adaptive molecular evolution predict the DFE for beneficial mutations is scale-free and exponentially distributed^6^,^24^. However, the available experimental data is conflicted^25^, and most studies have low statistical power due to the rare nature of beneficial mutations. While synthetically constructed, our competitive growth selection results yield fitness metrics for a large effective population size, and the hundreds of beneficial mutations observed provides high statistical power for model fitting. Predictions of beneficial DFE are derived from extreme value theory that describes many distributions falling under the umbrella of the generalized Pareto distribution (GPD)^24^. GPD includes three domains of attraction defined by their shape parameter (*κ*): Gumbel (*κ*=0), Fréchet (*κ*>0) and Weibull (*κ*<0). We first performed bootstrap goodness of fit tests to a GPD and concluded a failure to reject the null hypothesis that the data sets belonged to a GPD (*P*<0.066) and estimated *κ* to be −0.292, −0.309 and −0.195 for the ACT, PR and IB data sets, respectively. This finding indicates that the tail behaviour for the observed beneficial DFE for amiE is slightly truncated, yet our results are consistent with the predictions of Orr that if departures from the Gumbel domain are observed they will be minimal (−1/2<*κ*<1/2) (ref. 6).

We next conducted log-likelihood ratio tests for fitted exponential distributions (null hypothesis) against fitted gamma and Weibull distributions (alternative hypotheses, see Methods) for the DFE of beneficial mutations (Fig. 4b). These alternative models were chosen as previous empirical studies have observed tail behaviour indicative of these such distributions^40^,^45^,^46^. We concluded a failure to reject the null hypothesis for the IB data set, yet found that the ACT data set best fit a Weibull distribution (*P*=0.05) and that gamma and Weibull were both better fits for the PR data set (*P*=0.023 and 0.039, respectively, Table 1). Interestingly, one-sample Anderson-Darling tests for goodness-of-fit to each distribution indicated a failure to reject the null hypothesis that the data fit any of the distributions (Table 1). To assess the null hypothesis that the three data sets came from a single, statistically indistinguishable distribution, we performed a k-sample Anderson-Darling test and concluded they were not from a single distribution (*P*=0.0124). Thus, all data sets can be described as exponentially distributed, though the ACT and PR data sets best fit the higher parameter models.

### Beneficial mutations result mainly from protein effects

We addressed whether effects at the mRNA level could explain beneficial mutations, as variants can achieve higher fitness by increasing total active amiE concentration through improvements to the rate of transcription, the degradation rate of mRNA, and the efficiency of translation. The fitness metrics of synonymous codons for beneficial mutations (*ζ*>0.15) showed low variance in most cases except near the N terminus (Supplementary Fig. 7). A recent mRNA model^47^ could explain up to 5% of the variance in the first 15 residues but only 0.2% of the variance over the entire sequence length (Supplementary Table 4). We conclude that the observed fitness effects are mainly the result of changes at the protein level, not at the mRNA level.

### Comparison of DFE between selections

Promiscuous activity of enzymes is believed to be the driving force of evolution towards new activities^3^. Our fitness maps allow us to address the question of how mutations impact fitness in multiple substrate backgrounds. At the outset of this work, we anticipated that the majority of ‘hits' or beneficial mutations would be shared across selections. This null hypothesis is grounded in the biophysical argument that most beneficial mutations would improve protein expression, not activity, and these would be beneficial regardless of the substrate selected on. Additionally, we anticipated that the pool of mutations available for improving activity for a single substrate would predominately localize to the vicinity of the active site, thus rendering few specificity-altering mutations. Consequences of this prediction are that there should be significant correlation of fitness between different amides, with specificity-determining mutations encoded locally near the active site.

**Figure 5:**
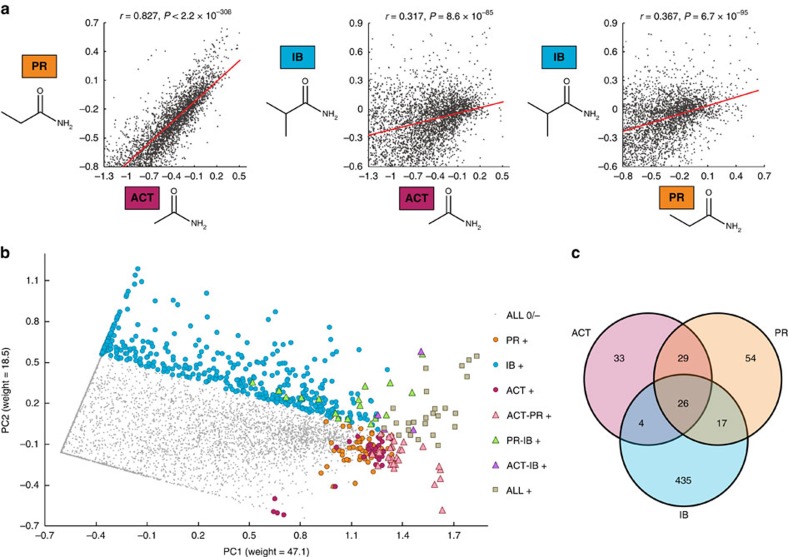
Correlative analysis of fitness effects. (**a**) Correlation of variant fitness metrics between selections. Variants with fitness metrics above the lower bounds are compared between each selection. Plots represents *n*=3,054, 3,600, and 2,959 points for panels ACT versus PR, ACT versus IB and PR versus IB, respectively. The reported *P* value for the Pearson's product moment correlation coefficient was calculated using a two-tailed *t*-test. (**b**) Principal component analysis of substrate-specific fitness effects. Black dots show common neutral and deleterious mutations, while substrate-specific beneficial mutations (*ζ*>0.15) are coloured according to seven bins. (**c**) 3-way Venn diagram representing seven specificity bins.

We first assessed whether there was a significant correlation of fitness between different amides (Fig. 5a). Correlation for the ACT and PR selections (*r*=0.827, *P*<2.2 × 10^−308^) was notably higher than that for the IB and ACT (*r*=0.317, *P*=8.6 × 10^−85^) or IB and PR selections (*r*=0.367, *P*=6.7 × 10^−95^). Principal component analysis revealed that a single principal component could explain 96.8% and 87.8% of the variance of the ACT and PR data sets, respectively, while two principal components are sufficient to explain over 99% of the variance for the IB data set (Fig. 5b, Supplementary Fig. 8). These results are inconsistent with our null hypothesis, pointing towards global alterations in the protein structure to adapt to different substrates.

Restricting our correlative analysis to only beneficial mutations (*ζ*>0.15) revealed that fitness-enhancing mutations for ACT were, on average, likely to be beneficial for PR (mean *ζ*=0.236). By contrast, beneficial mutations for IB were likely to be deleterious in both the ACT and PR selections (mean *ζ*=−0.480 and −0.319). This result is consistent with the findings of Stiffler *et al*.^38^ that beneficial mutations for a new or less evolved function are likely to be deleterious for existing functions when the selections pressures are high. Furthermore, IB-beneficial variants showed essentially no correlation for fitness in the ACT and PR selections (*r*=0.0617 and 0.164, respectively). This finding indicates that, at least for amiE, predicting hits based on known fitness effects for a given substrate cannot be accomplished through correlative analysis.

**Figure 6:**
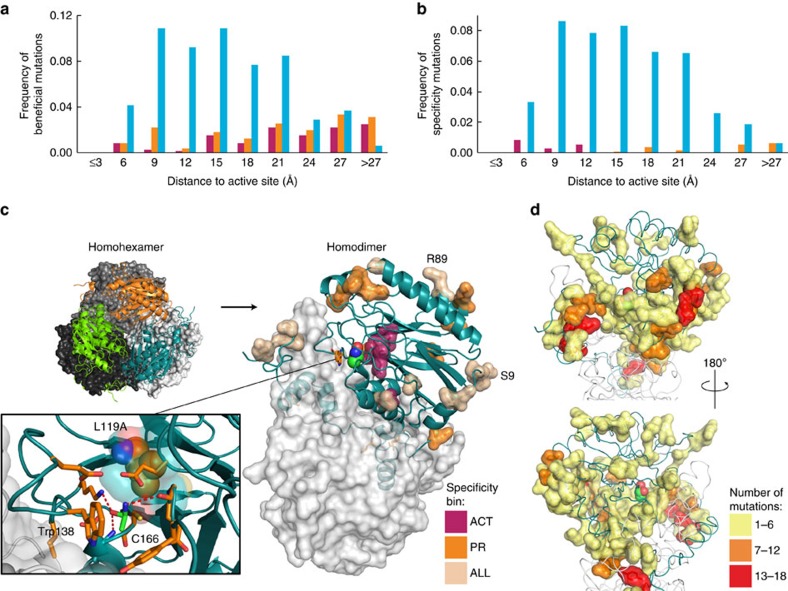
Substrate specificity is globally encoded. (**a**,**b**) Frequency of beneficial (**a**) and specificity-determining (**b**) mutations as a function of distance to active site. (**c**) Beneficial mutations for all selections and specificity-determinant mutations for ACT and PR selections mapped onto the structure of amiE. The inset illustrates the catalytic active site. (**d**) Specificity-determinant mutations for IB selection coloured by number found at given position.

We next analysed the relationship between beneficial (*ζ*>0.15) and specificity-determining (*ζ*>0.15 for one amide and *ζ*<0 for the other two substrates) mutations and their distance to the catalytic active site. Distance was measured by the minimum distance from the alpha-carbon of positions with beneficial mutations to any active site atom (six identical active sites in the functional homohexamer). The mutations were placed in 3 Å bins that were normalized to total available mutations in each distance shell. For beneficial mutations, we found that most were >15 Å from the active site for the ACT and PR variants, while the IB variants were mostly 9–21 Å away (Fig. 6a). Strikingly, we found very few specificity-determining mutations for the ACT and PR selections (*n*=6 and 14, respectively), with variants distanced by 6–15 Å for ACT and >14 Å for PR (Fig. 6b). By contrast, we found 395 specificity-determining mutations for IB, which were distributed similarly to the set of all IB-beneficial mutations. Thus, beneficial and specificity-determinant positions are globally dispersed throughout the primary sequence and structure of amiE.

### Biophysical characterization of beneficial mutations

To understand the biophysical basis underlying beneficial mutations, we expressed, purified, and characterized a set of 11 variants chosen in part on their ability to predict larger sets of beneficial variants (Table 2, Supplementary Fig. 9). For example, globally beneficial mutation S9A was chosen because it could potentially explain other N-terminal beneficial mutations. For all variants save one (see Methods), apparent melting temperatures (*T*_m,app_) were within 7 °C of the wild-type *T*_m,app_ of 67.7±0.1 °C, indicating that differences in thermal stability are unlikely to explain *in vivo* beneficial fitness effects.

To evaluate commonalities between beneficial mutations, we sorted variants into seven possible bins for beneficial fitness metrics (*ζ*>0.15 for given selection(s) and *ζ*<0.15 in other selection(s), Fig. 5c). Twenty-one of 26 beneficial mutations common to all three selections were found at extreme N- or C-terminal residues. Of the remaining five, we characterized R89E, a surface mutation located over 20 Å away from the active site that yielded an increase in relative *k*_cat_/*K*_m_ of 1.96±0.59 and 1.42±0.42 for PR and IB substrates, respectively (Fig. 6c). Alternatively, shared N-terminal mutation S9A had slightly reduced relative *k*_cat_/*K*_m_. Thus, even for a highly stable protein like amiE, we found few mutations like R89E that can generally increase *k*_cat_ or *K*_M_ and increase fitness.

Beneficial mutations shared in two of the three selections were scarce. In all, 18/29 mutations shared between ACT and PR cluster at the extreme N- or C- termini. The 17 PR+IB-specific mutations cluster at Q273, a 2nd shell residue that buttresses W138 at the active site and at M202 located 14 Å to the active site. Variant M202H showed over 2.5-fold increase in relative *k*_cat_/*K*_m_ for IB and PR, but Q273A did not show increased catalytic efficiency *in vitro*. We speculate the conditions required by the enzyme assay for sensitive ammonia detection prohibited the recapitulation of *in vivo* kinetics.

Four ACT-specific mutations encoded smaller substitutions (A/C/S/V) at position L119, a residue that supplies hydrophobic packing behind the catalytic nucleophile C166 10 Å from the active site (Fig. 6c). L119A showed a 2.2±0.1–fold increase in *k*_cat_ relative to wild-type with a compensatory increase in *K*_M_.

In stark contrast to ACT, there were 435 IB-specific and 395 specificity-determining mutations for IB distributed throughout the protein structure (Fig. 6d). Substitution W138A/G decreases van der Waals area in the vicinity of the amide transition state, allowing accommodation of the bulky isobutyrl group. However, most specificity-altering mutations were located far from the active site. Hot spots of positions where five or more specificity-determining mutations confer increased fitness occur at the N- and C-terminus (residues H3, S7, T323, R324, T327, V329 and C332-V334), as well as P50, C139, I174, A196, K197, V201, M202, W209, N212, F223, S228, G247-E249, G252, Q271, Q273-H275 and Y284. Interestingly, hot spot positions 197 through 212 are located on an alpha helix located at least 12 Å from the active site that contacts the dimeric interface. As these mutations do not benefit all substrates, we hypothesize that mutations at these positions cause rigid body motion of the helix to yield subtle geometric rearrangement, if not large-scale disruption, of the active site that favours IB catalysis. We tested V201M/T for activity on IB and, contrary to expectations, found a decrease in relative *k*_cat_/*K*_m_. We speculate that the mismatch between expected and measured catalytic efficiency results from hexamer dissociation caused by the low enzyme concentration required by the activity assay, as the lysate assays showed an increase in velocity for the V201T mutant.

## Discussion

In this contribution, we generated single-mutation protein fitness landscapes for an amidase on three different substrates. In contrast to studying protein–protein interactions, the application of deep mutational scanning to enzymes has been limited by the difficulty in developing generalizable high-throughput functional assays, as the nature of enzyme function is highly diverse. Regardless, exhaustive mutational studies permit a glimpse into how natural enzymes evolve for new functions. Our results show that, at least for amiE, mutations which are beneficial for only one substrate are (1) not confined to vicinity of the active site and (2) cannot be predicted based on known fitness for another substrate.

In terms of predicting fitness, we conclude that single-mutation fitness landscapes are highly substrate dependent, which is consistent with previous works^8^,^38^,^48^,^49^. However, this work provides a unique perspective of comparing two structurally similar substrates, ACT and PR, to the dissimilar IB substrate. Not surprisingly, we found the IB single-mutation fitness landscapes to be the most divergent, signalling that at the biophysical level the requirement to accommodate the larger IB substrate significantly alters the mutational landscape. The percentage of beneficial mutations observed is consistent with previous deep mutational scanning experiments on enzymes^8^,^37^,^38^,^50^. These rates are significantly larger than that predicted for the natural adaptation of organisms^51^ because in deep mutational scanning experiments a strong selection is imposed upon a gene that is, by experimental design, intended to influence only a single phenotypic trait^4^. This intention to mitigate pleiotropy is especially true with bulk growth competition experiments, and it should be noted that randomly drifting populations contain genes that do not ascribe to such constraints.

Our results strengthen the theoretical case that fitness for beneficial mutations is approximately exponentially distributed even though the percentage of beneficial mutations differs substantially between substrates. We note that this exponential distribution holds even for the IB selection which presumably causes large-scale rearrangements of the active site to allow better access to the branched chain IB. Other studies have explored the mechanics of multiple steps and epistasis^39^,^42^,^43^,^52^,^53^. In this work, we considered only single steps in the local fitness landscape. Thus, the generality of our observations for multiple steps remain to be seen.

For the design and engineering of substrate promiscuous or specific biocatalysts, knowledge of the sequence and spatial distribution of ‘hits' is imperative. Our findings indicate that, at least for amiE, most substrate-determining mutations for new functions, in this case IB, localize approximately 9–24 Å from the active site. Altogether, these results have strong implications for design and engineering of substrate promiscuous biocatalysts because it suggests current strategies of iterative site saturation mutagenesis near the active site are sub-optimal^1^. Additionally, computational design algorithms focused solely on the modifying 1st and 2nd shell mutations around the active site need to be revisited.

## Methods

### Reagents

All chemicals were purchased from Sigma-Aldrich unless specified otherwise. All primers and mutagenic oligonucleotides were designed using the Agilent QuikChange Primer Design Program (www.agilent.com) and were ordered from Integrated DNA Technologies. PR and IB solids were recrystallized from ethyl acetate and water, respectively.

### Plasmid construction

pEDA6_amiE was renamed from pJK_proK14_amiE as described in Bienick *et al*.^33^. pEDA2_amiE was constructed by Kunkel mutagenesis^54^ of pJK_proK17_amiE from Bienick *et al*.^33^ to introduce a knockdown ribosome binding sequence (primer kRBS3). Protein expression constructs were made by subcloning the *amiE* gene from pEDA2_amiE into the pET-29b(+) (Novagen) backbone at the *Nde*I and *Xho*I restriction sites following standard protocols. amiE point mutants were created using Kunkel mutagenesis^54^. Primer sequences used in this work are listed in Supplementary Table 5.

### Construction of mutagenesis libraries

Eight comprehensive single-site saturation mutagenesis libraries of amiE were constructed (residues 1–85, 86–170, 171–255 and 256–341 on plasmids pEDA2_amiE and pEDA6_amiE) using PFunkel mutagenesis^35^ with modifications as noted^34^. Library cell stocks of the selection strain, *E. coli* MG1655 rph+ [F- λ-] (Coli Genetic Stock Center, #7925), were made essentially as described in Klesmith *et al*.^50^.

### Growth selections

Starter cultures for growth selection were prepared as in Klesmith *et al*.^50^, except 1X M9 minimal media lacking ammonium chloride (M9 N^−^) was used to wash cell pellets before inoculation of selection media. Three millilitre of selection media (M9 N^−^ + 10 mM ACT, M9 N^−^ + 15 mM PR and M9 N^−^ + 10 mM IB for ACT, PR and IB selections, respectively) was inoculated to an initial OD_600_ of 0.02 at a volume of 3 ml (>6 × 10^6^ cells). To ensure exponential growth during the entire selection experiment, after approximately four generations the cells were harvested, washed with M9 N^−^, and a fresh 3 ml culture with selection media was inoculated to the same initial OD_600_ of 0.02 (to maintain the same initial population size). Growth selections were carried out and samples preserved for sequencing as described in Klesmith *et al*.^50^. Replicates were performed using the same pre-selection population. Based on the high correlation between replicates and the fact that a major source of error in deep sequencing measurements are counting errors (Poisson noise)^34^, the fitness metrics used in subsequent analysis were computed by combining reads from the two replicates and repeating the analysis.

### Sequencing

Libraries were amplified, barcoded, cleaned and quantified following Method B as described in Kowalsky *et al*.^34^. Gene amplification primers are listed in Supplementary Table 5. Pre- and post-selection samples were pooled and sequenced with 300 bp PE reads on an Illumina MiSeq available at the Michigan State University sequencing core. Deep sequencing data was analysed using Enrich software^55^ with modifications as noted in Kowalsky *et al*.^34^ and scripts freely available at Github (https://github.com/JKlesmith/Deep_Sequencing_Analysis).

Normalized fitness metrics for each variant, *ζ*_i_, were determined according to the ‘Normalization for Growth Rate Selections' section as outlined in Kowalsky *et al*.^34^. Briefly, deep sequencing was used to count each library member in the pre- and post-selection populations. For each single nonsynonymous mutation and wild-type an enrichment ratio was calculated by:


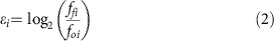


Where *f*_*fi*_ and *f*_*oi*_ represent the frequency of member *i* in the final (post-selection) and initial (pre-selection) populations. Normalized fitness metrics were calculated using the following equation:


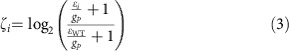


Where *ɛ*_*i*_ is enrichment ratio for variant i, *g*_*p*_ is the number of population doublings, and *ɛ*_WT_ is the enrichment ratio for wild-type.

### Beneficial mutations and lower bounds for fitness metrics

A beneficial mutation was defined as having at least 10% increase in growth rate (*ζ*>0.15) relative to wild-type. Weighted means for synonymous codon fitness metrics, where the weights were read counts (depth of coverage) for each mutation, were calculated to be 0.03±0.09, −0.02±0.11 and −0.01±0.07 for the ACT, PR and IB data sets, respectively. A fitness metric of 0.15 was found to be in >90% percentile for all three data sets.

To determine lower-bound fitness metrics for each selection, we first determined the half-median of read counts of the pre-selection library for each selection (63, 49 and 31 for the ACT, PR and IB selections, respectively). This number was normalized by the ratio of post- to pre-selection read counts (2.97, 1.86, and 2.04 for ACT, PR and IB selections, respectively). Next, a lower-bound enrichment ratio (*ɛ*_LB_) based on 10 read counts in the post-selection population was calculated:


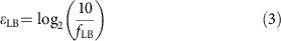


Where *f*_LB_ represents the normalized half-median pre-selection reads. The lower-bound fitness metrics, *ζ*_LB_, was then calculated using eight population doublings (*g*_p_) and the wild-type enrichment ratio (*ɛ*_WT_):


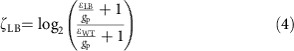


### Distribution fitting of beneficial DFE

Distribution fitting analysis was conducted using R statistical software^56^. Bootstrap goodness of fit and parameter estimation for the GPD were done using the package gPdtest^57^. Model parameters were approximated and log likelihood values were determined using maximum likelihood estimation with package fitdistrplus^58^. Anderson-Darling tests were performed using the package kSamples^59^. Log-likelihood (LL) ratios were calculated as 2*[(LL *H*_A_)–(LL *H*_0_)], where *H*_0_=null hypothesis and *H*_A_=alternative hypothesis. *P* values were computed from a chi-squared distribution with one degree of freedom.

### Protein characterization

Wild-type and variant amiE protein was expressed using Studier auto-induction^60^ and purified according to Klesmith *et al*.^50^. The eluate was buffer exchanged into PBS buffer, pH 7.5 using GE disposable PD-10 desalting columns (GE Healthcare). Purified protein was stored in PBS at 4 °C. Wild-type and variant amiE melting temperatures were measured using a SYPRO Orange thermal-shift assay^61^,^62^ as described in Klesmith *et al*.^50^, but in PBS buffer, pH 7.5. Catalytic parameters (*K*_m_ and *k*_cat_) were assayed at 37 °C in PBS buffer, pH 7.5 using a phenol and hypochlorite ammonia detection assay^63^. PCR plates containing 100 μl of seven different concentrations of amide (highest concentrations were 40, 150 and 800 mM for ACT, PR and IB activity assays, respectively, with 1:2 serial dilutions for remaining substrate concentrations) in PBS were incubated on a thermocyler block (Eppendorf) with the lid open at 37 °C for 5 min. To begin the assay, 20 μl of 0.02 μM (ACT and PR assays) or 0.2 μM (IB assays) enzyme was added. At discrete time points, 100 μl of the reaction was removed and quenched by depositing into a clear 96-well plate containing 50 μl phenol nitroprusside solution held on ice. At the end of the last time point, 50 μl alkaline hypochlorite solution was added to all wells and the plate was covered and incubated in a metal bead heat bath for 10 min at 35 °C. The plate was then transferred to a Synergy H1 spectrophotometer (BioTek) held at 35 °C and A_625_ was measured every minute for 15 min. Non-linear regression was performed using GraphPad Prism version 6 for Mac OS X, GraphPad Software, La Jolla California USA, www.graphpad.com. All measurements were performed at least in duplicate. The IB-specific variant M203W shows increased fitness in the deep-sequencing selection but decreased lysate activity compared with wild-type. M203W immediately precipitated out when we tried to purify this enzyme. Thus, for this case, lysate activity would not be representative of *in vivo* conditions. For PR variants, the coefficient of variation for wild-type was prohibitively high to calculate statistically significant ratios; note the variance of the other wild-types measurements.

### Isogenic growth and lysate flux assays

Starter cultures were prepared by inoculating 2 ml of M9 minimal media + carbenicillin (50 μg ml^−1^) with scrapings of MG1655 rph+ cell stocks harbouring pEDA2_amiE or pEDA6_amiE variant plasmids and grown overnight at 37 °C with 250 r.p.m. shaking. In the morning, cells were pelleted, washed twice with M9 N^−^, and resuspended in 1 ml M9 N^−^. 3 ml of selection media + carbenicillin (50 μg ml^−1^) in Hungate tubes was inoculated to a final OD_600_ of 0.02. Cultures were grown at 37 °C with shaking at 250 r.p.m. For growth assays, OD_600_ was measured every 30–45 min until a final OD_600_ of ∼0.5 was reached. All growth rate measurements represent at least four biological replicates collected on at least two separate dates. Lysate flux assays were adapted from Bienick *et al*.^33^. Two millilitre of exponential phase culture (OD_600_ of approximately 0.15–0.3) was spun down at 15,000*g* for 5 min. Cell pellets were washed twice and resuspended with 1 ml PBS, pH 7.5. Cells were lysed as described in Bienick *et al*.^33^. In all, 0.5–0.9 ml of lysate was used in a 1 ml total volume assay containing 10, 15 or 10 mM ACT, PR or IB, respectively. The assay was conducted at 37 °C. Every 5 min, 100 μl of the assay volume was removed and added to a 96-well plate containing 50 μl pre-chilled phenol nitroprusside. At the end of the last time point, 50 μl of alkaline hypochlorite was added to all wells. Absorbance at 625 nm was measured as in Bienick *et al*.^33^.

### Data availability

Full data sets including normalized fitness metrics, pre- and post-selection read counts, and raw log base two enrichment scores for each variant can be found in Supplementary Data 1-3 for the ACT, PR and IB selections, respectively, and can be obtained from Figshare (https://dx.doi.org/10.6084/m9.figshare.3505901.v2). Raw sequencing reads for this work have been deposited in the SRA (SAMN06237792-SAMN06237827).

## Additional information

**How to cite this article:** Wrenbeck, E. E. *et al*. Single-mutation fitness landscapes for an enzyme on multiple substrates reveal specificity is globally encoded. *Nat. Commun.* **8**, 15695 doi: 10.1038/ncomms15695 (2017).

**Publisher's note:** Springer Nature remains neutral with regard to jurisdictional claims in published maps and institutional affiliations.

## Supplementary Material

Supplementary Information Supplementary Figures, Supplementary Tables and Supplementary References

## Figures and Tables

**Table 1:** Model fitting results for distribution of beneficial mutations.

		**ACT**	**PR**	**IB**
*Exponential*	rate	8.72	6.67	7.26
	A-D test *P* value	0.527	0.338	0.635
	LL	365.9	303.2	1382.6
				
*Gamma*	shape	1.14	1.17	1.03
	rate	9.90	7.81	7.46
	A-D test *P* value	0.834	0.459	0.723
	LL	367.4	305.8	1382.9
				
*Weibull*	shape	1.094	1.094	1.012
	scale	0.119	0.155	0.138
	A-D test *P* value	0.851	0.451	0.712
	LL	367.8	305.3	1382.8
				
*Log-likelihood ratio tests*				
*H*_0_	*H*_A_			
Exponential	Gamma	3.11	5.14	0.62
	*P* value	0.078	0.023	0.43
Exponential	Weibull	3.8	4.3	0.31
	*P* value	0.050	0.039	0.58

**Table 2:** Wild-type and variant amiE biophysical data.

**Variant**	**ACT** ***ζ***	**PR** ***ζ***	**IB** ***ζ***	***K***_**m**_**(mM)/*****K***_**m,wt**_ **(mM)**	***k***_**cat**_ **(s**^−**1**^**) /*****k***_**cat,wt**_ **(s**^−**1**^**)**	***k***_**cat**_**/*****K***_**m**_ **(M**^−**1**^ **s**^−**1**^**) /*****k***_**cat,wt**_**/*****K***_**m,wt**_ **(M**^−**1**^ **s**^−**1**^**)**	***T***_**m,app**_ **(°C)**
Wild-type	0.00	0.00	0.00	4.7±0.552.7±8.3297.2±54.8	59.0±2.0144.7±9.913.3±1.1		67.7±0.1
							
S9A	0.33	0.41	0.36	2.3±0.64.4±1.10.5±0.1	0.6±0.11.6±0.20.4±0.0	0.28±0.10.36±0.130.76±0.27	63.1±0.1
							
A28R	0.27	0.10	0.11	nd	nd	nd	67.7±0.1
							
R89E	0.30	0.34	0.15	nd0.7±0.10.8±0.2	nd1.4±0.11.1±0.1	nd1.96±0.591.42±0.42	66.7±0.1
							
L119A	0.30	−0.80	−0.60	2.8±0.4Not active2.5±0.6	2.2±0.1Not active1.3±0.2	0.8±0.16Not active0.52±0.18	67.4±0.2
							
I165C	0.25	−0.27	−0.32	2.6±0.40.7±0.20.7±0.2	1.7±0.10.6±0.10.8±0.1	0.66±0.140.79±0.241.22±0.65	65.7±0.1
							
V201M	0.37	0.12	0.22	10.2±2.00.8±0.44.6 ±1.3	1.1±0.10.1±0.00.8±0.1	0.11±0.030.13±0.100.18±0.08	61.2±0.1
							
V201T	0.20	0.34	0.25	1.6±0.3nd3.8±0.8	1.0±0.1nd1.4±0.1	0.65±0.16nd0.37±0.11	64.9±0.2
							
M202H	−0.08	0.16	0.43	0.9±0.10.7±0.10.4±0.1	1.3±0.01.8±0.11.4±0.1	1.4±0.232.55±0.723.08±1.05	63.0±0.1
							
M203W	−1.30	−0.80	0.43	nd	nd	nd	nd
							
A234M	0.33	0.15	0.21	2.8±0.40.5±0.23.6±0.9	1.0±0.00.3±0.00.8±0.1	0.35±0.070.58±0.320.23±0.08	64.4±0.1
							
Q273A	−0.71	0.31	0.23	4.9±2.02.3±0.60.6±0.2	0.2±0.00.5±0.10.4±0.0	0.05±0.030.20±0.080.71±0.28	69.7±1.1
