# Supplementary material for: Single-mutation fitness landscapes for an enzyme on multiple substrates reveal specificity is globally encoded
Source: Nat Commun. 2017 Jun 6;8:15695. doi: 10.1038/ncomms15695 (PMC5467163; doi:10.1038/ncomms15695)
Supplement: Supplementary Information — Supplementary Figures, Supplementary Tables and Supplementary References [file ncomms15695-s1.pdf]

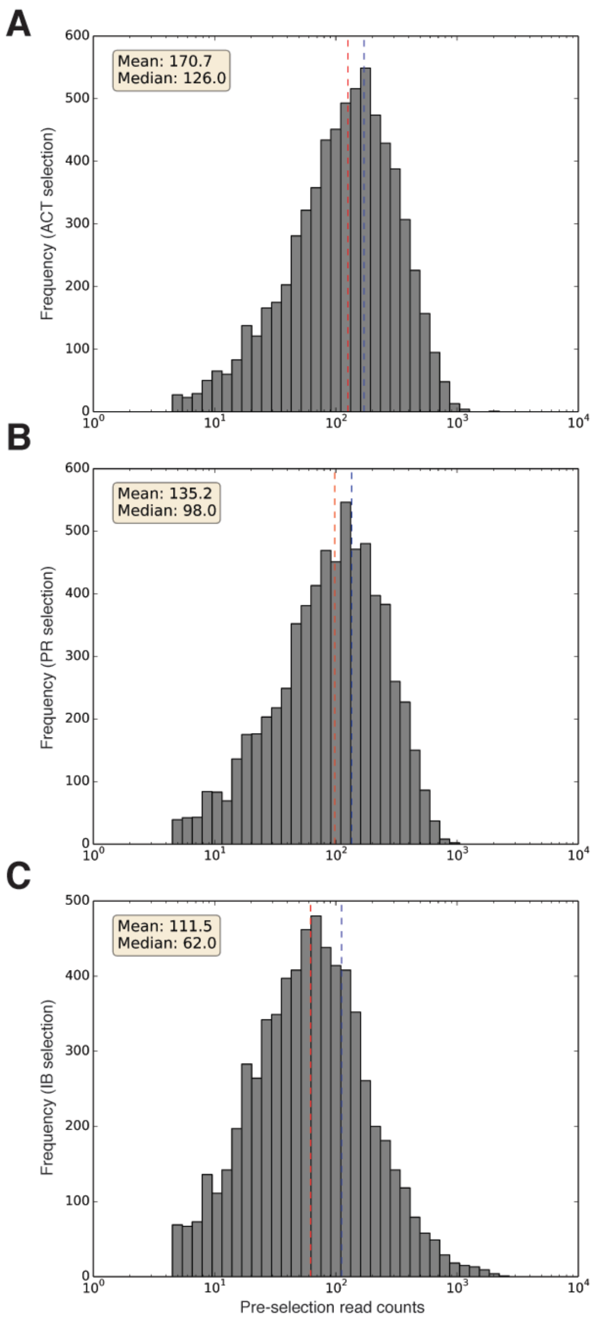

Supplementary Fig. 1

Frequency distribution of library member counts in the pre-selection libraries for A.) acetamide B.) propionamide and C.) isobutyramide. Vertical lines indicate median (red) and mean (blue) read coverage.

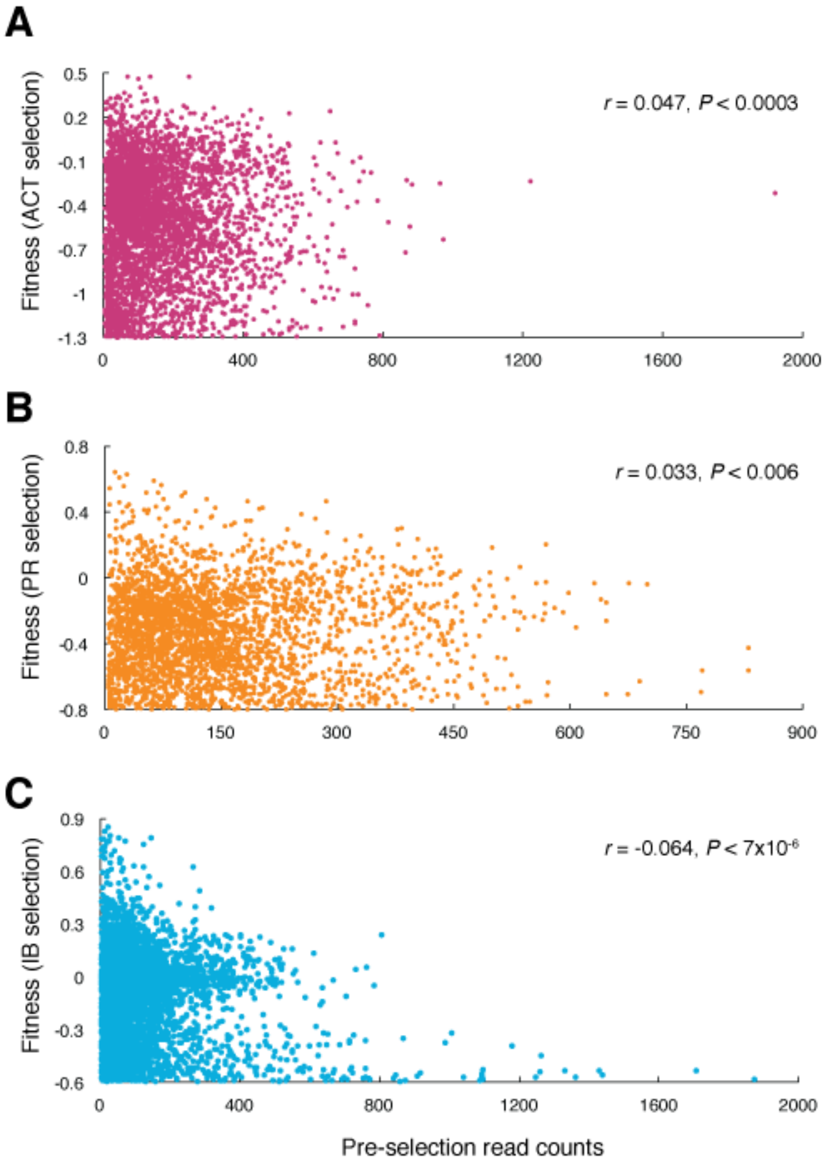

Supplementary Fig. 2

Fitness versus pre-selection read counts for each variant in the A.) acetamide B.) propionamide and C.) isobutyramide libraries. Variants with insignificant read counts ($n \leq 5$) and fitness metrics below the lower bounds were excluded from the analysis. Plots represent n = 4037, 3135, and 4969 variants. P-values for Pearson's product moment correlation coefficients were calculated using a two-tailed t-test.

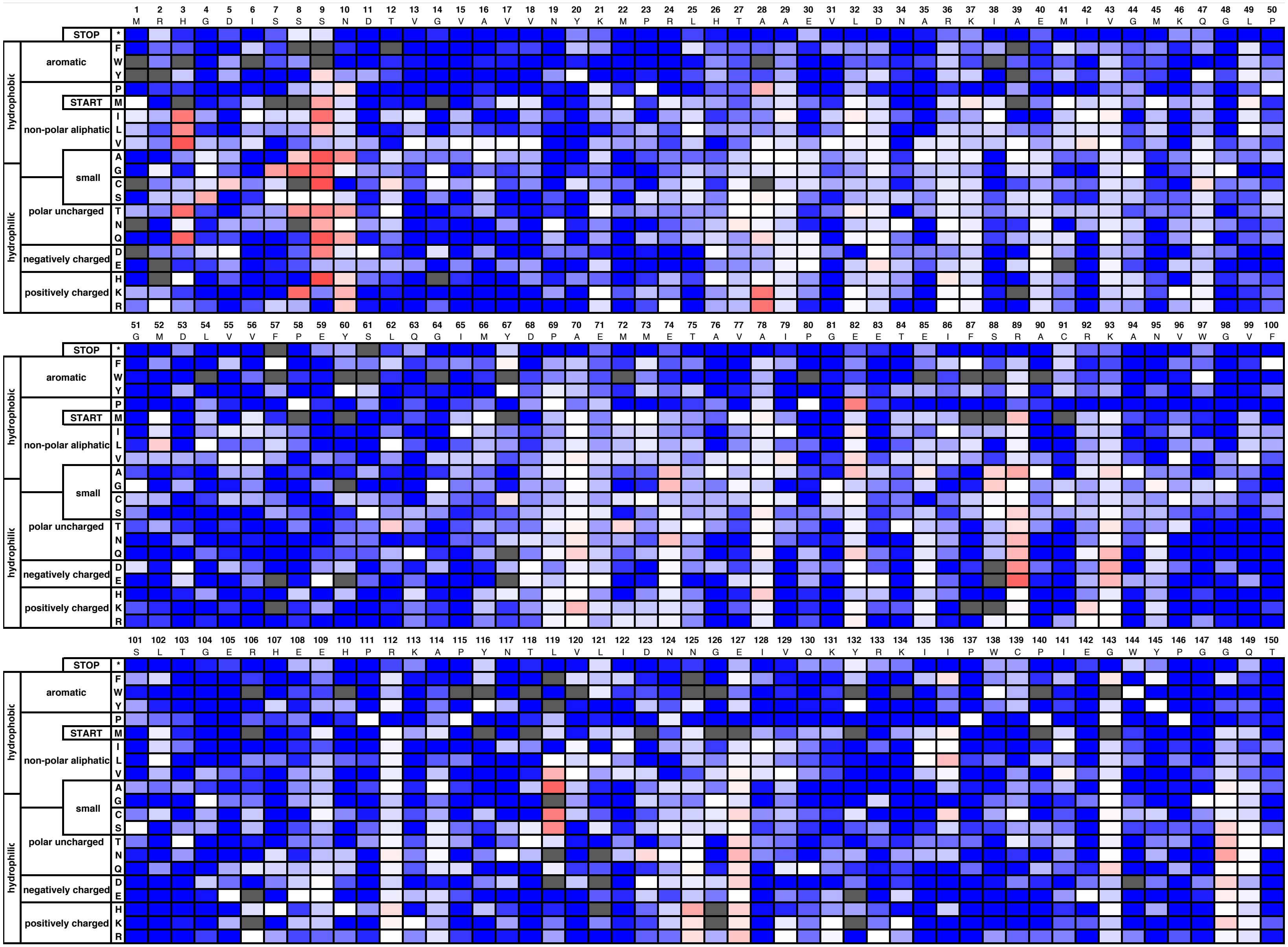
1 2 3 4 5 6 7 8 9 10 11 12 13 14 15 16 17 18 19 20 21 22 23 24 25 26 27 28 29 30 31 32 33 34 35 36 37 38 39 40 41 42 43 44 45 46 47 48 49 50
M R H G D I S S S N D T V G V A V V N Y K M P R L H T A A E V L D N A R K I A E M I V G M K Q G L P
51 52 53 54 55 56 57 58 59 60 61 62 63 64 65 66 67 68 69 70 71 72 73 74 75 76 77 78 79 80 81 82 83 84 85 86 87 88 89 90 91 92 93 94 95 96 97 98 99 100
G M D L V V F P E Y S L Q G I M Y D P A E M M E T A V A I P G E E T E I F S R A C R K A N V W G V F
101 102 103 104 105 106 107 108 109 110 111 112 113 114 115 116 117 118 119 120 121 122 123 124 125 126 127 128 129 130 131 132 133 134 135 136 137 138 139 140 141 142 143 144 145 146 147 148 149 150
S L T G E R H E E H P R K A P Y N T L V L I D N N G E I V Q K Y R K I I P W C P I E G W Y P G G Q T
STOP
START
hydrophobic
hydrophilic
aromatic
non-polar aliphatic
small
polar uncharged
negatively charged
positively charged
* F W Y P M I L V A G C S T N Q D E H K R

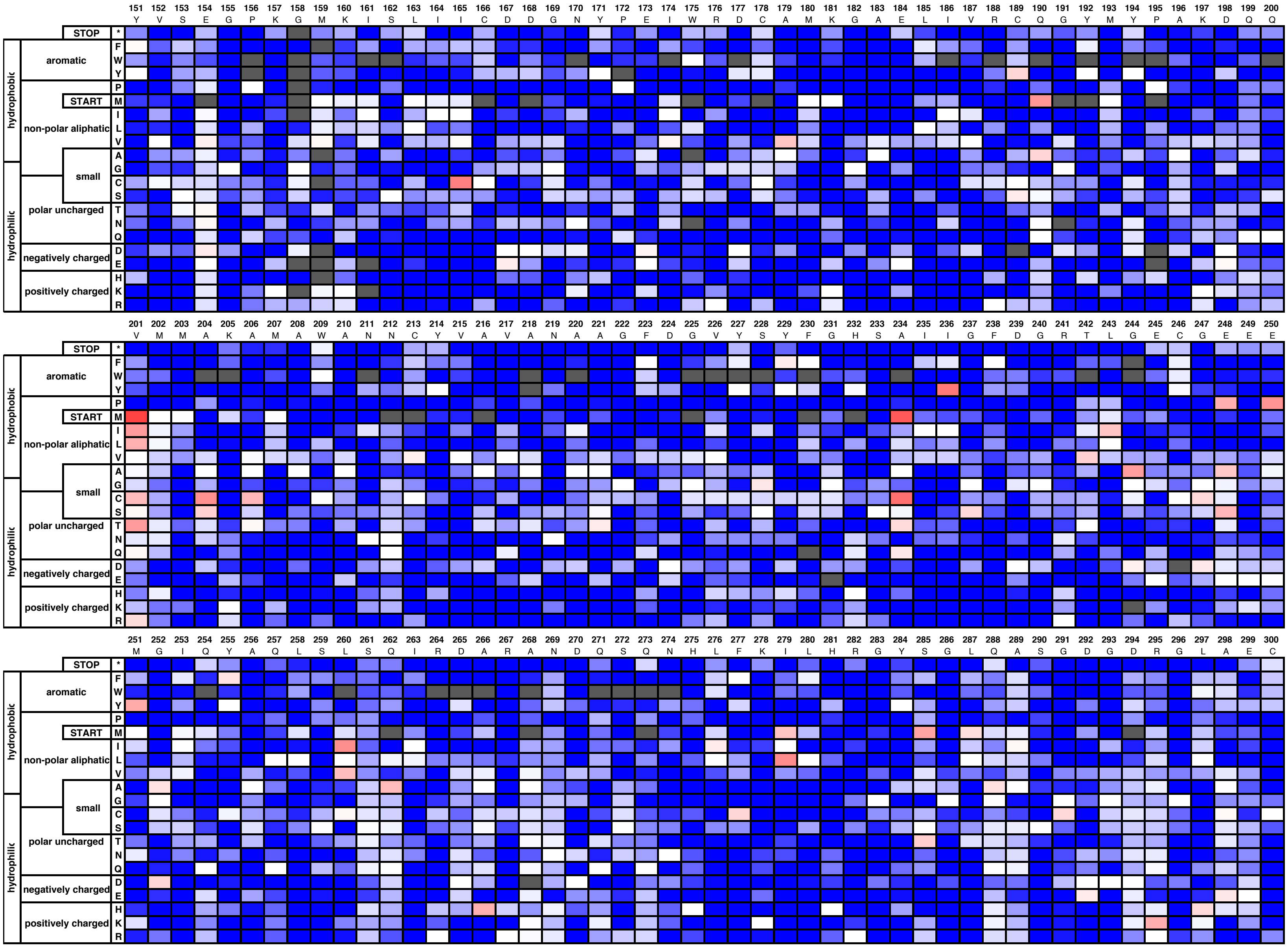
151 152 153 154 155 156 157 158 159 160 161 162 163 164 165 166 167 168 169 170 171 172 173 174 175 176 177 178 179 180 181 182 183 184 185 186 187 188 189 190 191 192 193 194 195 196 197 198 199 200
Y V S E G P K G M K I S L I I C D D G N Y P E I W R D C A M K G A E L I V R C Q G Y M Y P A K D Q Q
201 202 203 204 205 206 207 208 209 210 211 212 213 214 215 216 217 218 219 220 221 222 223 224 225 226 227 228 229 230 231 232 233 234 235 236 237 238 239 240 241 242 243 244 245 246 247 248 249 250
V M M A K A M A W A N N C Y V A V A N A A G F D G V Y S Y F G H S A I I G F D G R T L G E C G E E E
251 252 253 254 255 256 257 258 259 260 261 262 263 264 265 266 267 268 269 270 271 272 273 274 275 276 277 278 279 280 281 282 283 284 285 286 287 288 289 290 291 292 293 294 295 296 297 298 299 300
M G I Q Y A Q L S L S Q I R D A R A N D Q S Q N H L F K I L H R G Y S G L Q A S G D G D R G L A E C
STOP *
hydrophobic
hydrophilic
aromatic F W Y
P
START M
non-polar aliphatic I L V
small A G C S
polar uncharged T N Q
negatively charged D E
positively charged H K R

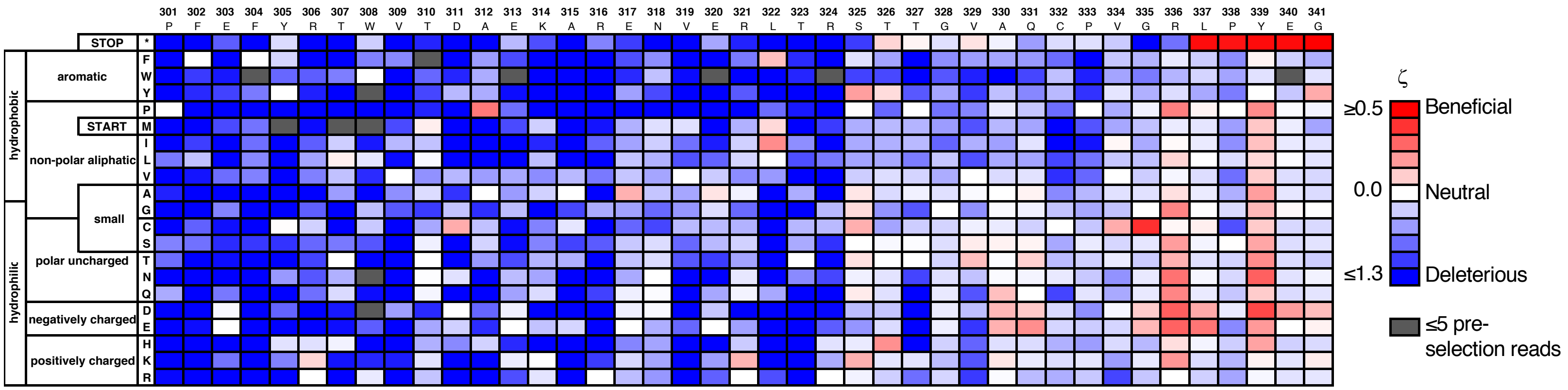

Supplementary Fig. 3

Fitness landscape for acetamide selection.

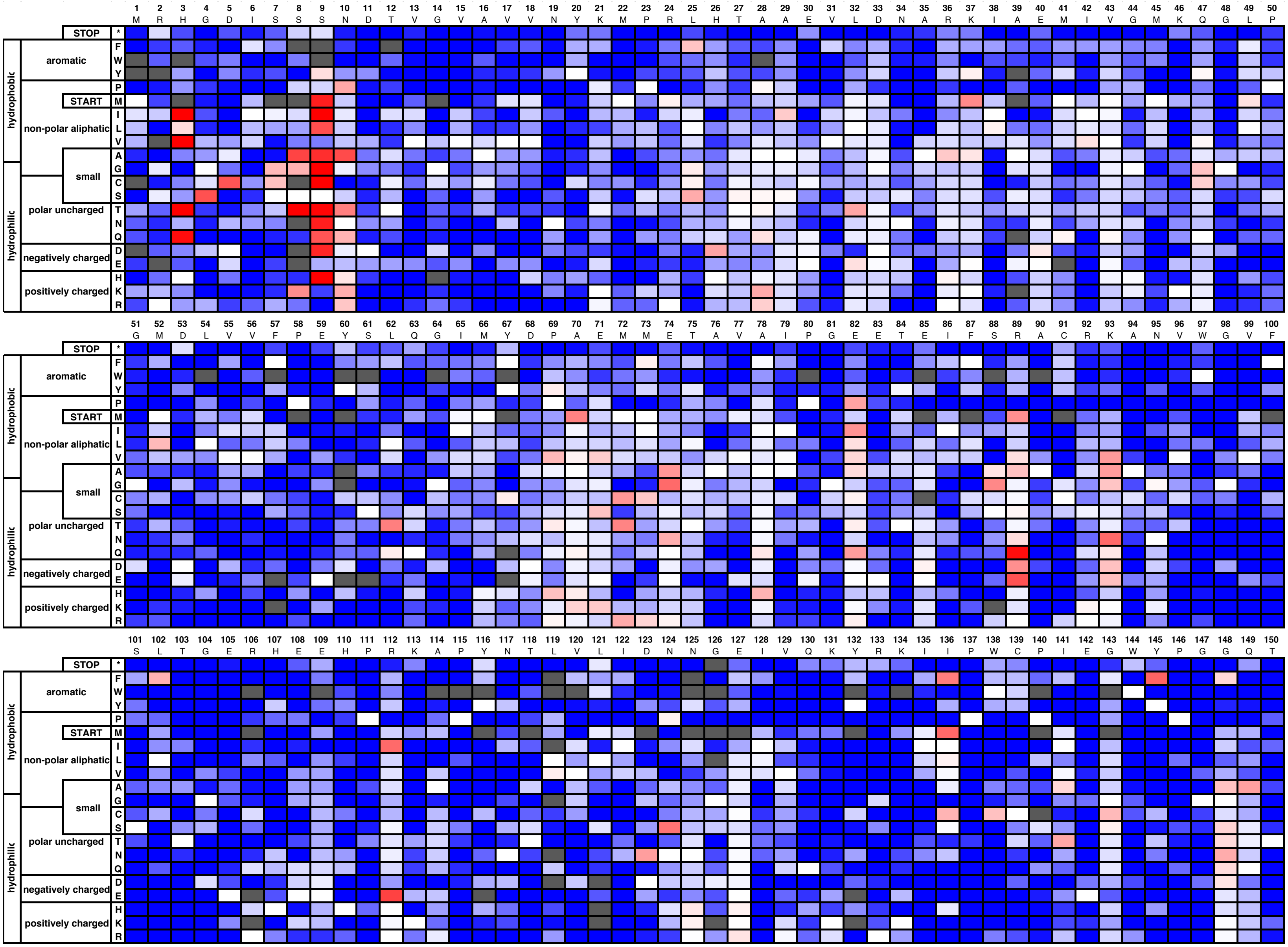
1 2 3 4 5 6 7 8 9 10 11 12 13 14 15 16 17 18 19 20 21 22 23 24 25 26 27 28 29 30 31 32 33 34 35 36 37 38 39 40 41 42 43 44 45 46 47 48 49 50
M R H G D I S S S N D T V G V A V V N Y K M P R L H T A A E V L D N A R K I A E M I V G M K Q G L P
51 52 53 54 55 56 57 58 59 60 61 62 63 64 65 66 67 68 69 70 71 72 73 74 75 76 77 78 79 80 81 82 83 84 85 86 87 88 89 90 91 92 93 94 95 96 97 98 99 100
G M D L V V F P E Y S L Q G I M Y D P A E M M E T A V A I P G E E T E I F S R A C R K A N V W G V F
101 102 103 104 105 106 107 108 109 110 111 112 113 114 115 116 117 118 119 120 121 122 123 124 125 126 127 128 129 130 131 132 133 134 135 136 137 138 139 140 141 142 143 144 145 146 147 148 149 150
S L T G E R H E E H P R K A P Y N T L V L I D N N G E I V Q K Y R K I I P W C P I E G W Y P G G Q T
STOP
*
hydrophobic
aromatic
F
W
Y
P
START
M
non-polar aliphatic
I
L
V
small
A
G
C
S
hydrophilic
polar uncharged
T
N
Q
negatively charged
D
E
positively charged
H
K
R

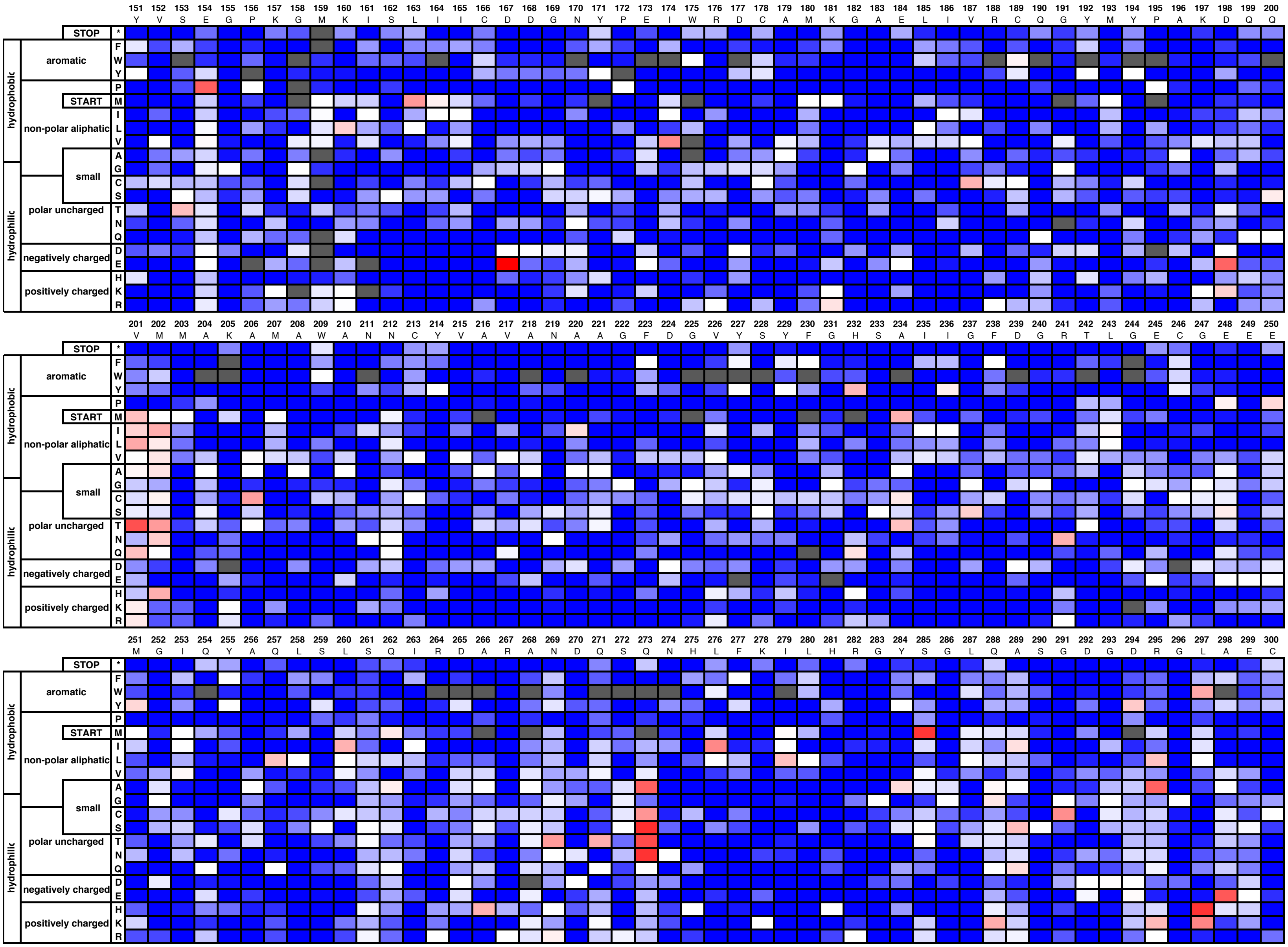
151 152 153 154 155 156 157 158 159 160 161 162 163 164 165 166 167 168 169 170 171 172 173 174 175 176 177 178 179 180 181 182 183 184 185 186 187 188 189 190 191 192 193 194 195 196 197 198 199 200
Y V S E G P K G M K I S L I I C D D G N Y P E I W R D C A M K G A E L I V R C Q G Y M Y P A K D Q Q
201 202 203 204 205 206 207 208 209 210 211 212 213 214 215 216 217 218 219 220 221 222 223 224 225 226 227 228 229 230 231 232 233 234 235 236 237 238 239 240 241 242 243 244 245 246 247 248 249 250
V M M A K A M A W A N N C Y V A V A N A A G F D G V Y S Y F G H S A I I G F D G R T L G E C G E E E
251 252 253 254 255 256 257 258 259 260 261 262 263 264 265 266 267 268 269 270 271 272 273 274 275 276 277 278 279 280 281 282 283 284 285 286 287 288 289 290 291 292 293 294 295 296 297 298 299 300
M G I Q Y A Q L S L S Q I R D A R A N D Q S Q N H L F K I L H R G Y S G L Q A S G D G D R G L A E C
STOP
START
hydrophobic
hydrophilic
aromatic
non-polar aliphatic
small
polar uncharged
negatively charged
positively charged
* F W Y P M I L V A G C S T N Q D E H K R

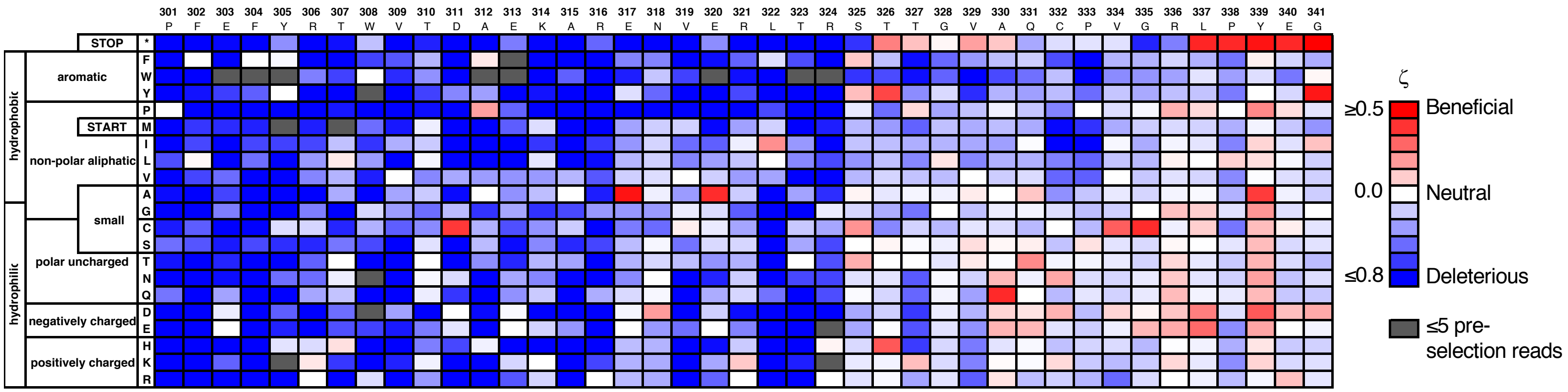

Supplementary Fig. 4

Fitness landscape for propionamide selection.

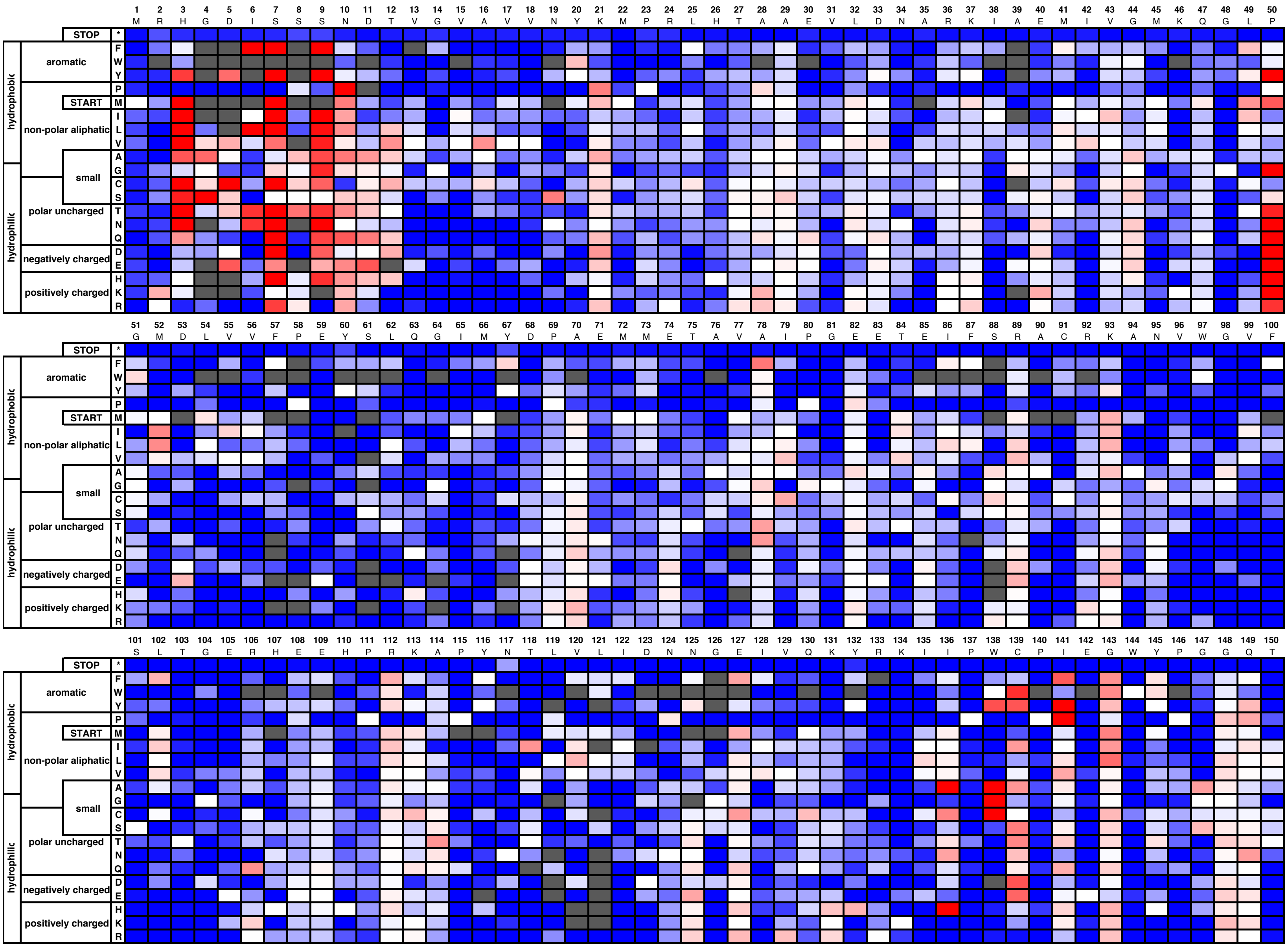

1 2 3 4 5 6 7 8 9 10 11 12 13 14 15 16 17 18 19 20 21 22 23 24 25 26 27 28 29 30 31 32 33 34 35 36 37 38 39 40 41 42 43 44 45 46 47 48 49 50
M R H G D I S S S N D T V G V A V V N Y K M P R L H T A A E V L D N A R K I A E M I V G M K Q G L P
51 52 53 54 55 56 57 58 59 60 61 62 63 64 65 66 67 68 69 70 71 72 73 74 75 76 77 78 79 80 81 82 83 84 85 86 87 88 89 90 91 92 93 94 95 96 97 98 99 100
G M D L V V F P E Y S L Q G I M Y D P A E M M E T A V A I P G E E T E I F S R A C R K A N V W G V F
101 102 103 104 105 106 107 108 109 110 111 112 113 114 115 116 117 118 119 120 121 122 123 124 125 126 127 128 129 130 131 132 133 134 135 136 137 138 139 140 141 142 143 144 145 146 147 148 149 150
S L T G E R H E E H P R K A P Y N T L V L I D N N G E I V Q K Y R K I I P W C P I E G W Y P G G Q T
STOP
*
aromatic
F W Y
P
START
M
non-polar aliphatic
I L V
small
A G C S
polar uncharged
T N Q
negatively charged
D E
positively charged
H K R
hydrophobic
hydrophilic

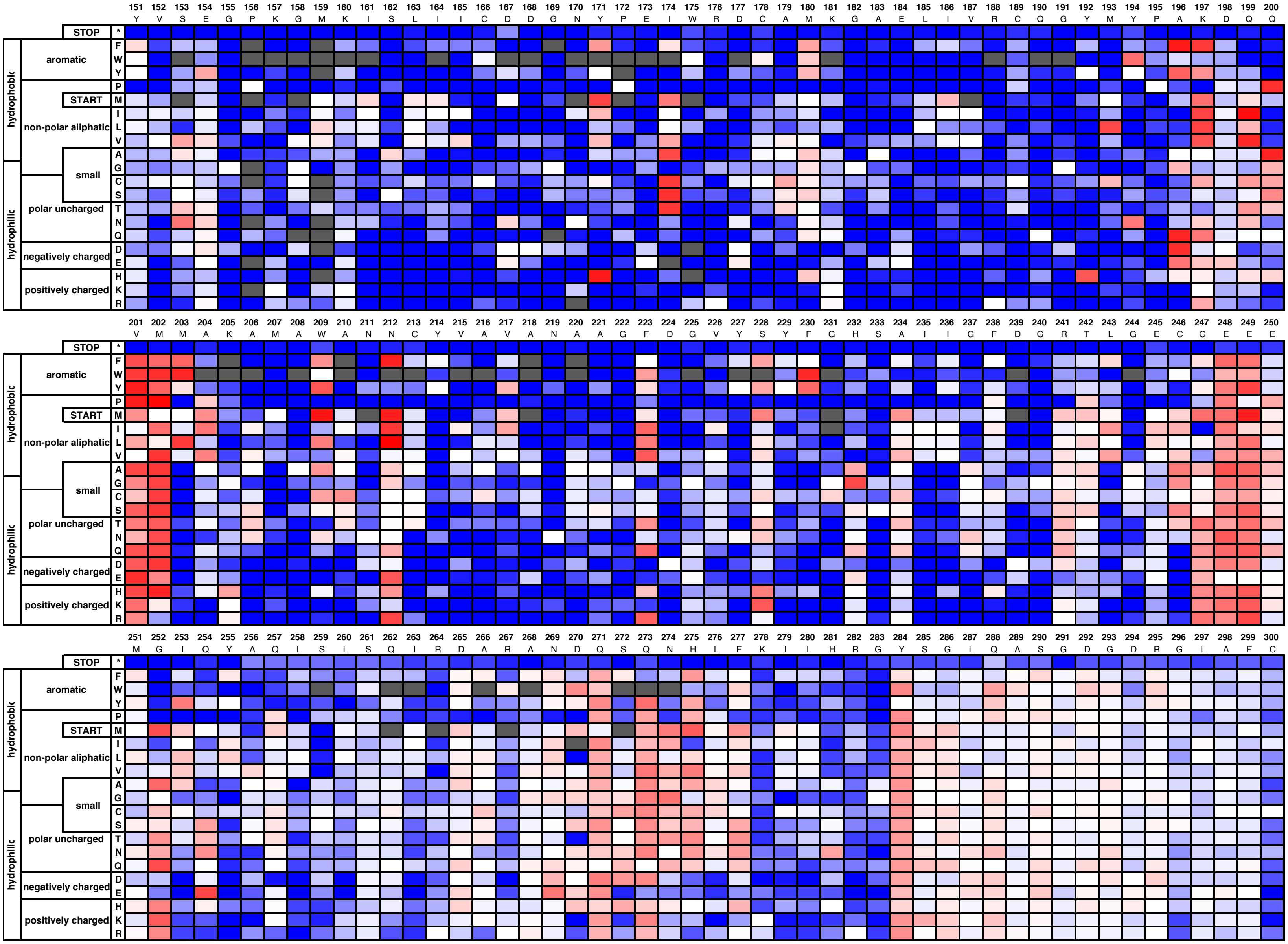
151 152 153 154 155 156 157 158 159 160 161 162 163 164 165 166 167 168 169 170 171 172 173 174 175 176 177 178 179 180 181 182 183 184 185 186 187 188 189 190 191 192 193 194 195 196 197 198 199 200
Y V S E G P K G M K I S L I I C D D G N Y P E I W R D C A M K G A E L I V R C Q G Y M Y P A K D Q Q
201 202 203 204 205 206 207 208 209 210 211 212 213 214 215 216 217 218 219 220 221 222 223 224 225 226 227 228 229 230 231 232 233 234 235 236 237 238 239 240 241 242 243 244 245 246 247 248 249 250
V M M A K A M A W A N N C Y V A V A N A A G F D G V Y S Y F G H S A I I G F D G R T L G E C G E E E
251 252 253 254 255 256 257 258 259 260 261 262 263 264 265 266 267 268 269 270 271 272 273 274 275 276 277 278 279 280 281 282 283 284 285 286 287 288 289 290 291 292 293 294 295 296 297 298 299 300
M G I Q Y A Q L S L S Q I R D A R A N D Q S Q N H L F K I L H R G Y S G L Q A S G D G D R G L A E C
STOP *
hydrophobic
aromatic F W Y
P
START M
non-polar aliphatic I L V
small A G C S
hydrophilic
polar uncharged T N Q
negatively charged D E
positively charged H K R

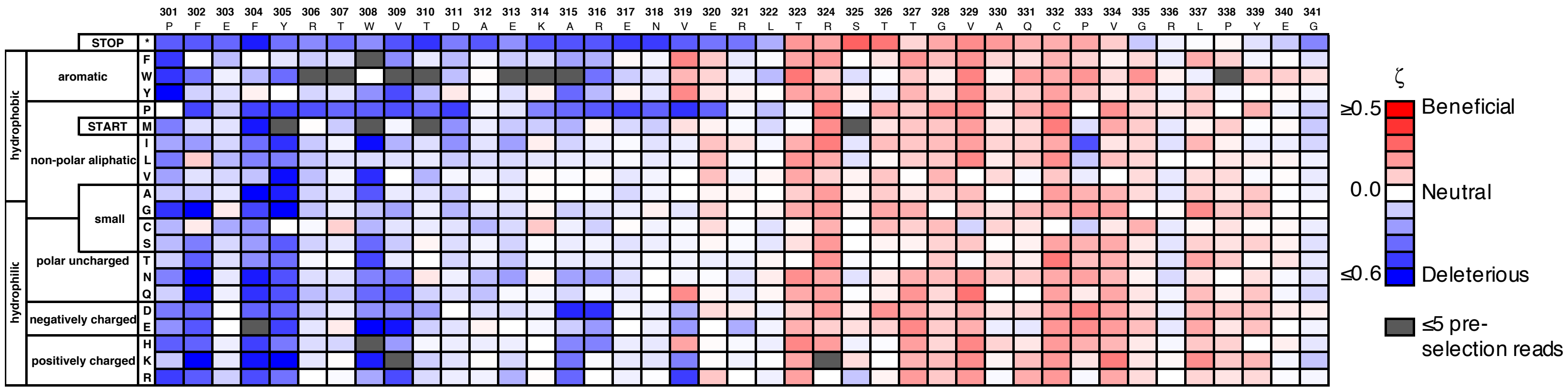

Supplementary Fig. 5

Fitness landscape for isobutyramide selection.

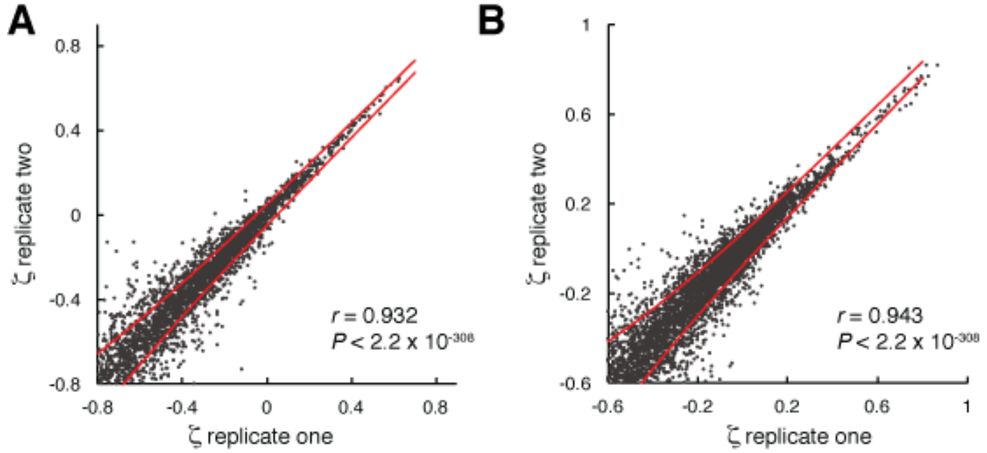

Supplementary Fig. 6
Fitness metrics from biological replicate growth selection experiments in A.) acetamide and B.) isobutyramide media. Plots represent n = 3834 and 4977 variants for panels A and B, respectively. Red lines indicate two standard deviations from theoretical error estimation[1]. P-values for Pearson's product moment correlation coefficients were calculated using a two-tailed t-test.

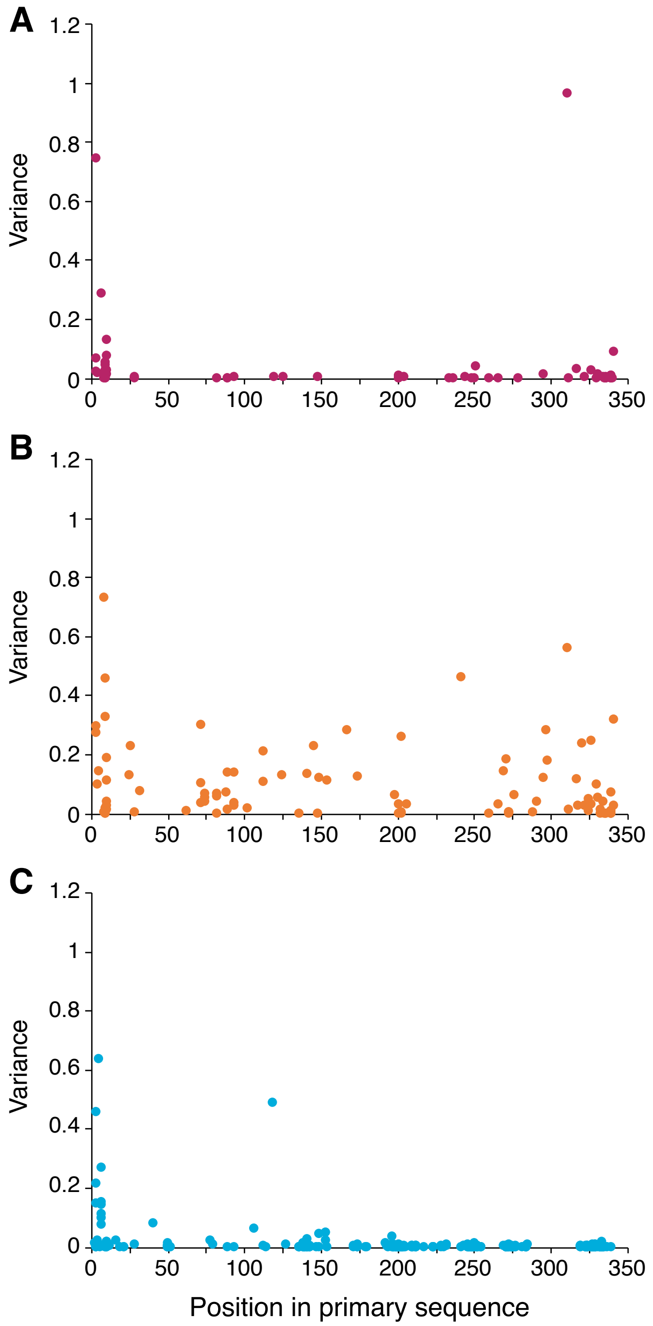

Supplementary Fig. 7

Variance of fitness metrics for synonymous codons of beneficial mutations (ζ>0.15) for the A.) acetamide B.) propionamide and C.) isobutyramide selections as a function of position in the primary sequence.

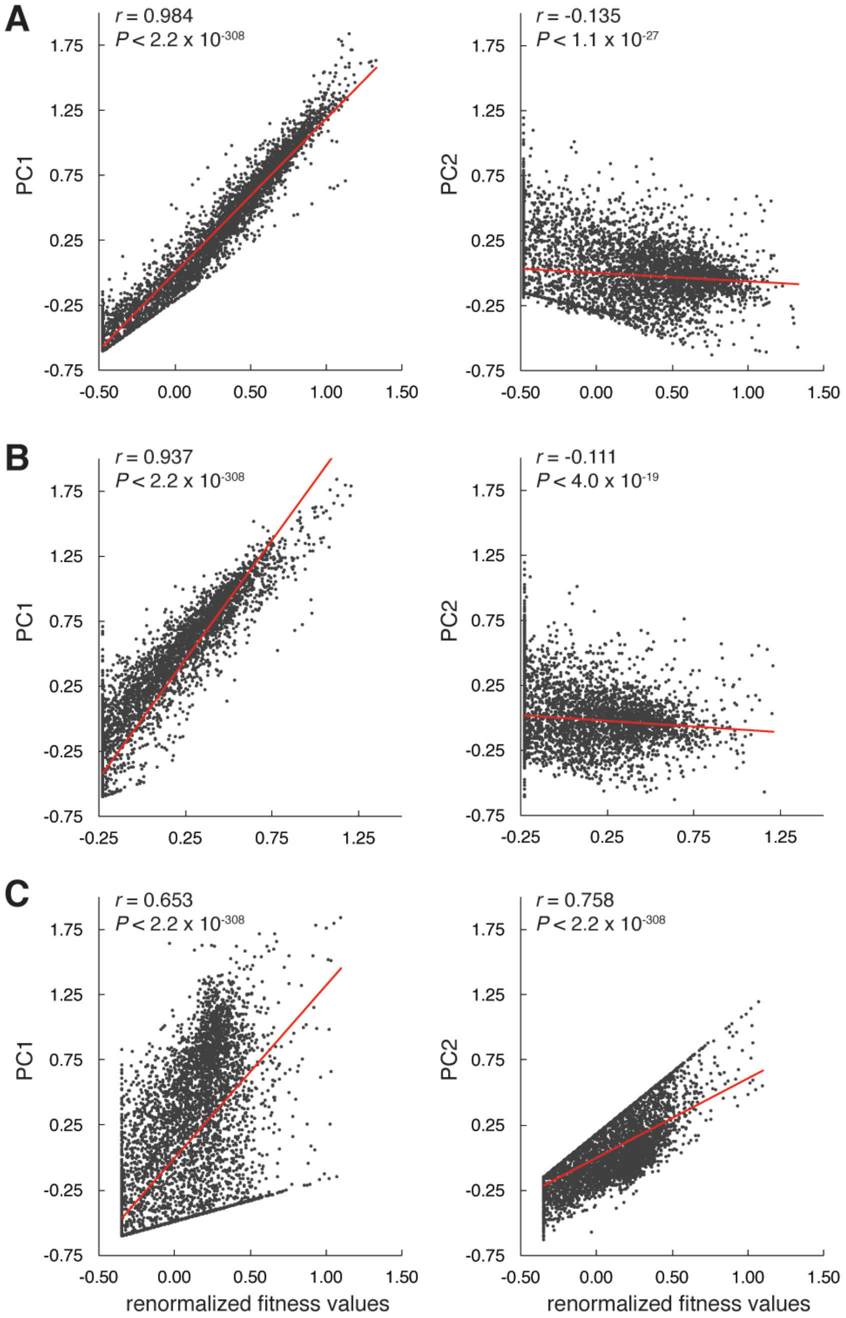

Supplementary Fig. 8

Principle component analysis of renormalized fitness values for A.) acetamide B.) propionamide and C.) isobutyramide selections. Fitness values were renormalized by subtracting the mean fitness (mean = -0.824, -0.575, -0.255 for acetamide, propionamide, and isobutyramide, respectively) from each variant. P-values for Pearson's product moment correlation coefficients were calculated using a two-tailed t-test.

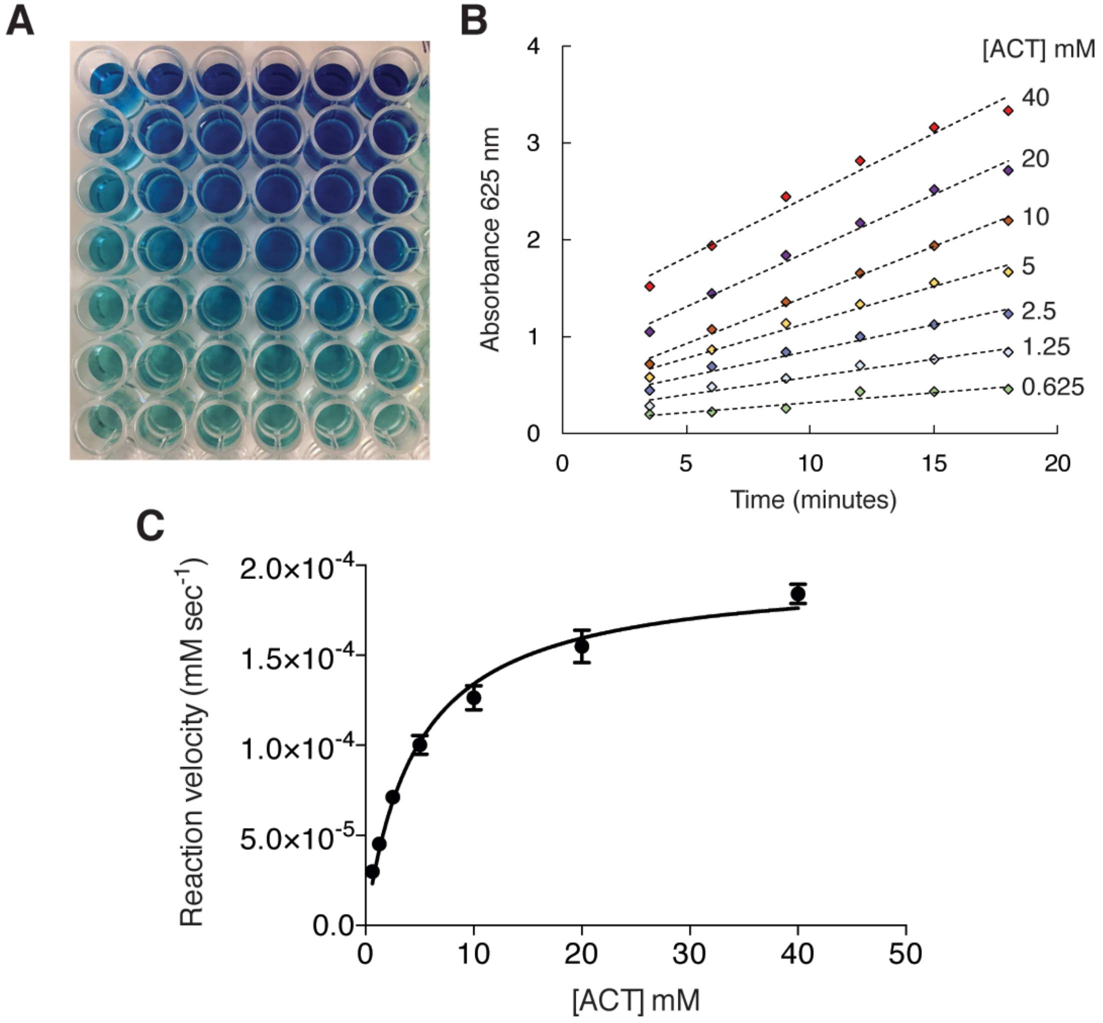

Supplementary Fig. 9

amiE activity assay. A.) amiE activities were measured using a colorimetric Berthelot reaction[2,3] for ammonia detection with phenol nitroprusside and alkaline hypochlorite. B.) Representative data for the amiE activity assay. Absorbance at 625 nm was measured at discrete time intervals for reactions containing one of seven concentrations of acetamide (ACT) and purified wild-type amiE. Reaction velocities were calculated by obtaining the slopes of each line. C.) Michaelis-Menten plot of wild-type amiE activity on acetamide substrate. Plot represents four independent measurements. Non-linear regression was performed using GraphPad Prism version 6 for Mac OS X, GraphPad Software, La Jolla California USA, www.graphpad.com.

Supplementary Table 1.

Constructs used in growth selections. ACT, PR, and IB = acetamide, propionamide, and isobutyramide selection media (see **Methods**). Promoters obtained from Bienick et al.[3].

plasmid	selection media	promoter	-35 hexamer	-10 hexamer	RBS name	RBS sequence	$\mu_{S,wt}$ (hr^{-1})<br>$\mu_{M9,wt}$ (hr^{-1})<br>$\mu_{S,wt}/\mu_{M9,wt}$
pJK_proK17_amiE	**ACT**	proK17	TTCCCG	TAATAT	t7RBS	AGGAGA	0.60 ± 0.02<br>0.65 ± 0.03<br>**0.92 ± 0.05**
pEDA2_amiE	**ACT**	proK17	TTCCCG	TAATAT	kRBS3	AGTTTT	0.44 ± 0.04<br>0.78 ± 0.03<br>**0.56 ± 0.06**
pEDA2_amiE	**PR**	proK17	TTCCCG	TAATAT	kRBS3	AGTTTT	0.29 ± 0.06<br>0.78 ± 0.03<br>**0.37 ± 0.08**
pEDA6_amiE	**IB**	proK14	TGTACG	TAATAT	t7RBS	AGGAGA	0.36 ± 0.07<br>0.66 ± 0.04<br>**0.54 ± 0.11**

Supplementary Table 2.

Library coverage statistics for combined amiE libraries (replicate 1 and 2) used in the acetamide, propionamide, and isobutyramide selections. Raw sequencing reads were quality filtered using Enrich[4].

	**Acetamide selection**	**Propionamide selection**	**Isobutyramide selection**
**Pre-selection population DNA reads post quality filter**	1,935,216	1,735,919	1,390,991
**Post-selection population DNA reads post quality filter**	7,738,122	3,236,744	2,831,599
**Percent of possible codon substitutions observed:**			
1-base substitution	100.0	100.0	100.0
2-base substitution	97.6	97.6	98.5
3-base substitution	95.7	95.6	97.2
All substitutions	97.1	97.1	98.1
**Percent of reads in pre-selection library with:**			
No nonsynonymous mutations	40.0	39.6	38.3
One nonsynonymous mutation	52.0	51.8	52.6
Multiple nonsynonymous mutations	8.0	8.7	9.1
**Coverage of possible single nonsynonymous mutations:**	**97.2**	**97.2**	**96.3**

Supplementary Table 3.

Isogenic growth and lysate flux data. Confidence intervals in error are given as 1 s.d. of at least 3 independent measurements.

variant	fitness metric (selection)	μ_i (hr^{-1})	μ_i/μ_{WT}	theoretical μ_i/μ_{WT}	lysate flux J_i/J_{WT} (mmol NH_3 $gDCW^{-1}$ hr^{-1})
wildtype	0.00 (ACT)	0.44 ± 0.04	1.00	1.00	0.15 ± 0.02
wildtype	0.00 (PR)	0.29 ± 0.06	1.00	1.00	*
wildtype	0.00 (IB)	0.36 ± 0.07	1.00	1.00	0.11 ± 0.01
S9A	0.33 (ACT)	0.68 ± 0.04	1.40 ± 0.08	1.26	nd
A28R	0.27 (ACT)	0.59 ± 0.03	1.44 ± 0.07	1.21	nd
L119A	0.30 (ACT)	0.66 ± 0.01	1.35 ± 0.03	1.23	1.98 ± 0.66
I136A	-0.07 (PR)	0.26 ± 0.00	0.78 ± 0.01	0.95	nd
I136A	0.52 (IB)	0.58 ± 0.00	1.31 ± 0.01	1.43	nd
I136H	-0.10 (PR)	0.33 ± 0.01	0.96 ± 0.02	0.94	nd
Q149A	0.19 (PR)	0.25 ± 0.00	0.88 ± 0.01	1.14	nd
I165C	0.25 (ACT)	0.62 ± 0.03	1.51 ± 0.08	1.19	nd
Y192V	-1.3 (ACT)	ng	nd		nd
V201M	0.12 (PR)	0.37 ± 0.01	1.09 ± 0.03	1.09	nd
V201M	0.22 (IB)	0.50 ± 0.00	1.29 ± 0.07	1.17	nd
V201T	0.20 (ACT)	0.52 ± 0.01	1.07 ± 0.02	1.15	2.08 ± 0.66
V201T	0.34 (PR)	0.39 ± 0.00	1.17 ± 0.01	1.27	nd
V201T	0.25 (IB)	0.56 ± 0.01	1.25 ± 0.02	1.19	1.9 ± 0.23
M203W	0.43 (IB)	0.61 ± 0.01	1.86 ± 0.07	1.34	0.69 ± 0.07
A234M	0.33 (ACT)	0.63 ± 0.01	1.29 ± 0.03	1.25	nd
A234M	0.15 (PR)	0.42 ± 0.01	1.25 ± 0.02	1.11	nd
A234M	0.21 (IB)	0.50 ± 0.01	1.14 ± 0.02	1.15	nd
I236Y	0.26 (ACT)	0.60 ± 0.00	1.22 ± 0.02	1.20	nd
Q273A	0.23 (IB)	0.59 ± 0.01	1.55 ± 0.09	1.17	nd

*error in measurements was prohibitively high for calculating ratios

nd = not determined

ng = no growth

Supplementary Table 4.

Pearson correlation analysis of mRNA model parameters calculated as in[5] with codon fitness metrics obtained in this work. Analysis was restricted to variants with ≥50 pre-selection read counts.

			All codons		Codons 2-16	
		Term	*r*	p-value	*r*	p-value
ACT		# of variants	6760		196	
	mRNA folding parameters	ΔG_{UH}	-0.022	0.073	-0.111	0.122
		a_H	0.008	0.492	0.069	0.338
		g_H	-0.003	0.814	-0.003	0.966
		u_{3H}	0.012	0.325	0.163	0.022
		**$\pi(\theta_{wt})$**	**-0.022**	**0.069**	**0.219**	**0.002**
	Codon influence parameters	$\Sigma\beta_c f_c$	-0.033	0.007	0.301	0.000
		$s_{7\text{-}16}$	0.031	0.010	0.247	0.000
		$s_{17\text{-}32}$	-0.020	0.092	-	-
		r	0.023	0.061	0.084	0.240
PR		# of variants	6193		161	
	mRNA folding parameters	ΔG_{UH}	-0.0293	0.021	-0.1421	0.071
		a_H	0.0036	0.777	0.0625	0.429
		g_H	-0.0131	0.303	-0.0641	0.418
		u_{3H}	-0.0061	0.630	0.1002	0.205
		**$\pi(\theta_{wt})$**	**-0.0131**	**0.303**	**0.2256**	**0.004**
	Codon influence parameters	$\Sigma\beta_c f_c$	-0.0087	0.496	0.2795	0.000
		$s_{7\text{-}16}$	0.0245	0.054	0.3097	0.000
		$s_{17\text{-}32}$	-0.0147	0.248	-	-
		r	0.0131	0.304	0.1038	0.189
IB		# of variants	2975		43	
	mRNA folding parameters	ΔG_{UH}	0.015	0.407	-0.025	0.874
		a_H	0.097	0.000	0.275	0.071
		g_H	-0.066	0.000	-0.161	0.297
		u_{3H}	0.088	0.000	0.162	0.295
		**$\pi(\theta_{wt})$**	**0.044**	**0.017**	**0.064**	**0.678**
	Codon influence parameters	$\Sigma\beta_c f_c$	-0.048	0.008	-0.224	0.143
		$s_{7\text{-}16}$	0.028	0.125	-0.303	0.045
		$s_{17\text{-}32}$	-0.016	0.392	-	-
		r	-0.015	0.402	-0.012	0.936

Supplementary Table 5.

Gene amplification primers used to prepare samples for deep sequencing.

**Gene amplification: inner primers**	
Fwd_Tile1_amiE	gttcagagttctacagtccgacgatcttaactttaagaagtttttatacat
Fwd_Tile1_amiE-2	gttcagagttctacagtccgacgatcttaactttaagaaggagatatacat
Fwd_Tile2_amiE	gttcagagttctacagtccgacgatcggcgaagaaacggaa
Fwd_Tile3_amiE	gttcagagttctacagtccgacgatcctgcgatgacggtaat
Fwd_Tile4_amiE	gttcagagttctacagtccgacgatcaagaaatgggcattcaatac
Rev_Tile1_amiE	ccttggcacccgagaattccaaagcacggctaaagat
Rev_Tile2_amiE	ccttggcacccgagaattccactctccaaatttccggata
Rev_Tile3_amiE	ccttggcacccgagaattccacagagacaactgcgc
Rev_Tile4_amiE	ccttggcacccgagaattccatggtggtgctcgag
blue = Illumina sequencing primer; black = gene overlap	
**Gene amplification: outer primers**	
Illumina_FWD	aatgatacggcgaccaccgagatctacacgttcagagttctacagtccga
**Primer (selection, sample)**	
RPI41 (ACT, T1-1)	caagcagaagacggcatacgagat**GTCGTC**gtgactggagttccttggcacccgagaattcca
RPI38 (ACT, T1-2)	caagcagaagacggcatacgagat**AGCTAG**gtgactggagttccttggcacccgagaattcca
RPI33 (ACT, T2-1)	caagcagaagacggcatacgagat**CGCCTG**gtgactggagttccttggcacccgagaattcca
RPI34 (ACT, T2-2)	caagcagaagacggcatacgagat**GCCATG**gtgactggagttccttggcacccgagaattcca
RPI43 (ACT, T3-1)	caagcagaagacggcatacgagat**GCTGTA**gtgactggagttccttggcacccgagaattcca
RPI40 (ACT, T3-2)	caagcagaagacggcatacgagat**TCTGAG**gtgactggagttccttggcacccgagaattcca
RPI44 (ACT, T4-1)	caagcagaagacggcatacgagat**ATTATA**gtgactggagttccttggcacccgagaattcca
RPI41 (ACT, T4-2)	caagcagaagacggcatacgagat**GTCGTC**gtgactggagttccttggcacccgagaattcca
RPI37 (ACT, T1U)	caagcagaagacggcatacgagat**ATTCCG**gtgactggagttccttggcacccgagaattcca
RPI22 (ACT, T2U)	caagcagaagacggcatacgagat**CGTACG**gtgactggagttccttggcacccgagaattcca
RPI39 (ACT, T3U)	caagcagaagacggcatacgagat**GTATAG**gtgactggagttccttggcacccgagaattcca
RPI40 (ACT, T4U)	caagcagaagacggcatacgagat**TCTGAG**gtgactggagttccttggcacccgagaattcca
RPI25 (PR, T1-1)	caagcagaagacggcatacgagat**ATCAGT**gtgactggagttccttggcacccgagaattcca
RPI26 (PR, T1-2)	caagcagaagacggcatacgagat**GCTCAT**gtgactggagttccttggcacccgagaattcca
RPI27 (PR, T2-1)	caagcagaagacggcatacgagat**AGGAAT**gtgactggagttccttggcacccgagaattcca
RPI28 (PR, T2-2)	caagcagaagacggcatacgagat**CTTTTG**gtgactggagttccttggcacccgagaattcca
RPI29 (PR, T3-1)	caagcagaagacggcatacgagat**TAGTTG**gtgactggagttccttggcacccgagaattcca
RPI30 (PR, T3-2)	caagcagaagacggcatacgagat**CCGGTG**gtgactggagttccttggcacccgagaattcca
RPI31 (PR, T4-1)	caagcagaagacggcatacgagat**ATCGTG**gtgactggagttccttggcacccgagaattcca
RPI32 (PR, T4-2)	caagcagaagacggcatacgagat**TGAGTG**gtgactggagttccttggcacccgagaattcca
RPI21 (PR, T1U)	caagcagaagacggcatacgagat**CGAAAC**gtgactggagttccttggcacccgagaattcca

RPI22 (PR, T2U)	caagcagaagacggcatacgagat**CGTACG**gtgactggagttccttggcacccgagaattcca
RPI23 (PR, T3U)	caagcagaagacggcatacgagat**CCACTC**gtgactggagttccttggcacccgagaattcca
RPI24 (PR, T4U)	caagcagaagacggcatacgagat**GCTACC**gtgactggagttccttggcacccgagaattcca
RPI13 (IB, T1-1)	caagcagaagacggcatacgagat**TTGACT**gtgactggagttccttggcacccgagaattcca
RPI14 (IB, T1-2)	caagcagaagacggcatacgagat**GGAACT**gtgactggagttccttggcacccgagaattcca
RPI15 (IB, T2-1)	caagcagaagacggcatacgagat**TGACAT**gtgactggagttccttggcacccgagaattcca
RPI16 (IB, T2-2)	caagcagaagacggcatacgagat**GGACGG**gtgactggagttccttggcacccgagaattcca
RPI17 (IB, T3-1)	caagcagaagacggcatacgagat**CTCTAC**gtgactggagttccttggcacccgagaattcca
RPI18 (IB, T3-2)	caagcagaagacggcatacgagat**GCGGAC**gtgactggagttccttggcacccgagaattcca
RPI19 (IB, T4-1)	caagcagaagacggcatacgagat**TTTCAC**gtgactggagttccttggcacccgagaattcca
RPI20 (IB, T4-2)	caagcagaagacggcatacgagat**GGCCAC**gtgactggagttccttggcacccgagaattcca
RPI9 (IB, T1U)	caagcagaagacggcatacgagat**CTGATC**gtgactggagttccttggcacccgagaattcca
RPI10 (IB, T2U)	caagcagaagacggcatacgagat**AAGCTA**gtgactggagttccttggcacccgagaattcca
RPI11 (IB, T3U)	caagcagaagacggcatacgagat**GTAGCC**gtgactggagttccttggcacccgagaattcca
RPI12 (IB, T4U)	caagcagaagacggcatacgagat**TACAAG**gtgactggagttccttggcacccgagaattcca
red = Illumina adapter sequence; **BOLD** = barcode; blue = Illumina sequencing primer	

Supplementary References

1. Kowalsky, C. A. *et al.* High-Resolution Sequence-Function Mapping of Full-Length Proteins. *PLoS One* **10,** e0118193 (2015).
2. Searle, P. L. The Berthelot or indophenol reaction and its use in the analytical chemistry of nitrogen. A review. *Analyst* **109,** 549–568 (1984).
3. Bienick, M. S. *et al.* The Interrelationship between Promoter Strength, Gene Expression, and Growth Rate. *PLoS One* **9,** e109105 (2014).
4. Fowler, D. M., Araya, C. L., Gerard, W. & Fields, S. Enrich: Software for analysis of protein function by enrichment and depletion of variants. *Bioinformatics* **27,** 3430–3431 (2011).
5. Boël, G. *et al.* Codon influence on protein expression in E . coli correlates with mRNA levels. *Nature* **529,** 358–363 (2016).
